# Supplementary material for: Therapeutic Potential of Beaucarnea recurvata Leaf Extract Against Ulcerative Colitis: Integrating Phytochemical Profiling, Network Pharmacology, and Experimental Validation
Source: Int J Mol Sci. 2025 Dec 15;26(24):12053. doi: 10.3390/ijms262412053 (PMC12733345; doi:10.3390/ijms262412053)
Supplement: Supplementary file 1 [file ijms-26-12053-s001.zip › Figure S1.docx]

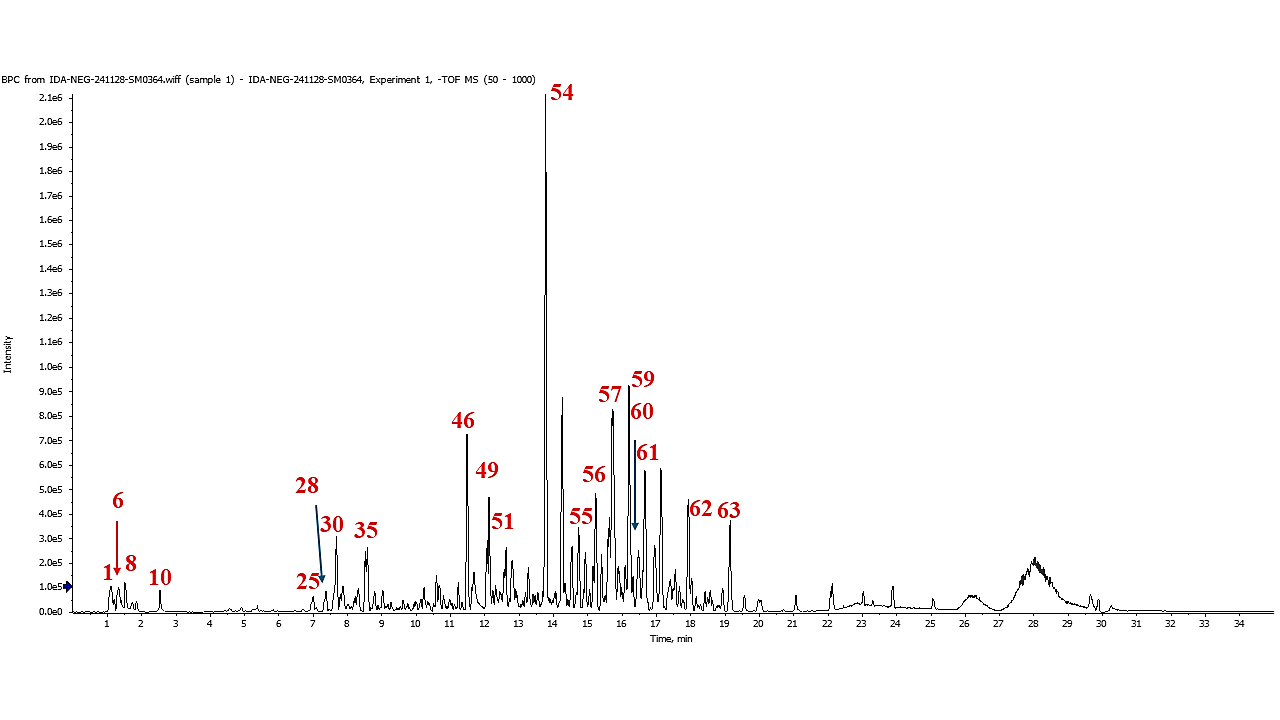


**Figure S1.** UPLC-ESI-MS/MS total ion chromatogram of *Beaucarnea recurvata* leaf extract in negative ion mode.
