# Supplementary material for: Therapeutic Potential of Beaucarnea recurvata Leaf Extract Against Ulcerative Colitis: Integrating Phytochemical Profiling, Network Pharmacology, and Experimental Validation
Source: Int J Mol Sci. 2025 Dec 15;26(24):12053. doi: 10.3390/ijms262412053 (PMC12733345; doi:10.3390/ijms262412053)
Supplement: Supplementary file 1 [file ijms-26-12053-s001.zip › Figure S2-S3.docx]

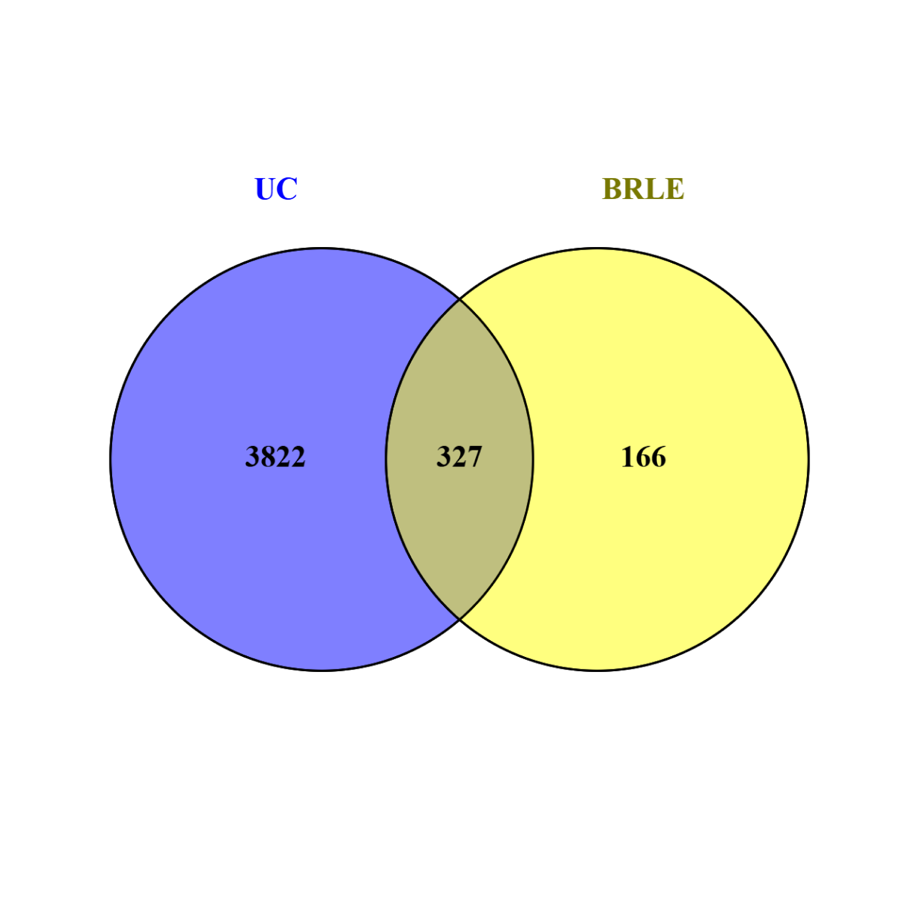


**Figure S2.** Intersection of molecular targets between ulcerative colitis (UC) pathogenesis and bioactive con-stituents from Beaucarnea recurvata leaf extract (BRLE).


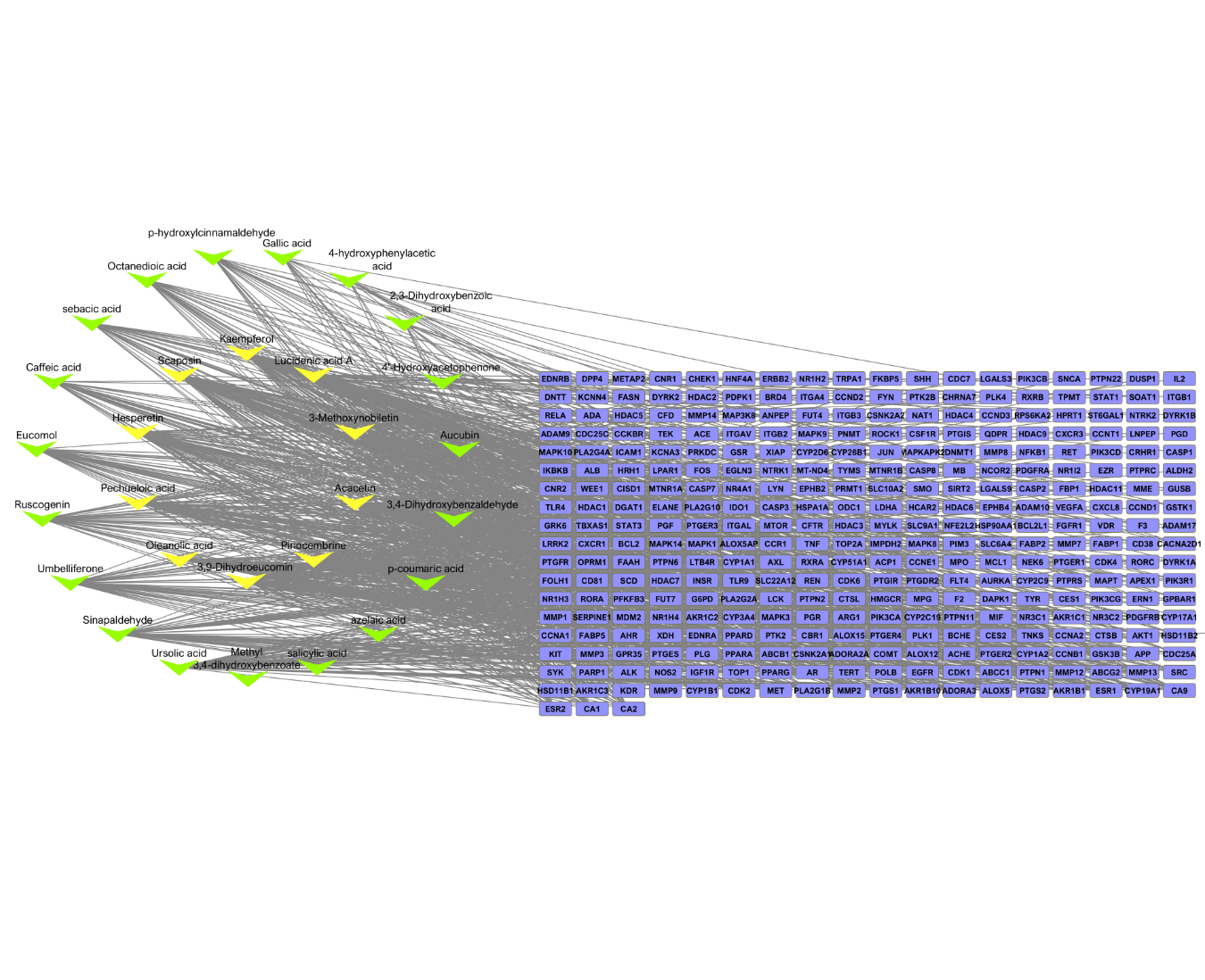


**Figure S3.** Compound-target interaction network for *Beaucarnea recurvata* leaf extract. High-connectivity compounds are represented as yellow arrows, low-connectivity compounds as green arrows, and potential therapeutic targets as blue rectangles.
