# Supplementary material for: Therapeutic Potential of Beaucarnea recurvata Leaf Extract Against Ulcerative Colitis: Integrating Phytochemical Profiling, Network Pharmacology, and Experimental Validation
Source: Int J Mol Sci. 2025 Dec 15;26(24):12053. doi: 10.3390/ijms262412053 (PMC12733345; doi:10.3390/ijms262412053)
Supplement: Supplementary file 1 [file ijms-26-12053-s001.zip › Figure S4-S7.docx]

| **Oleanolic acid** | |
| --- | --- |
| **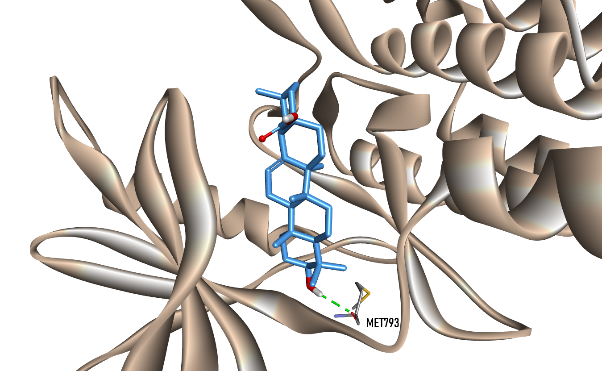** | **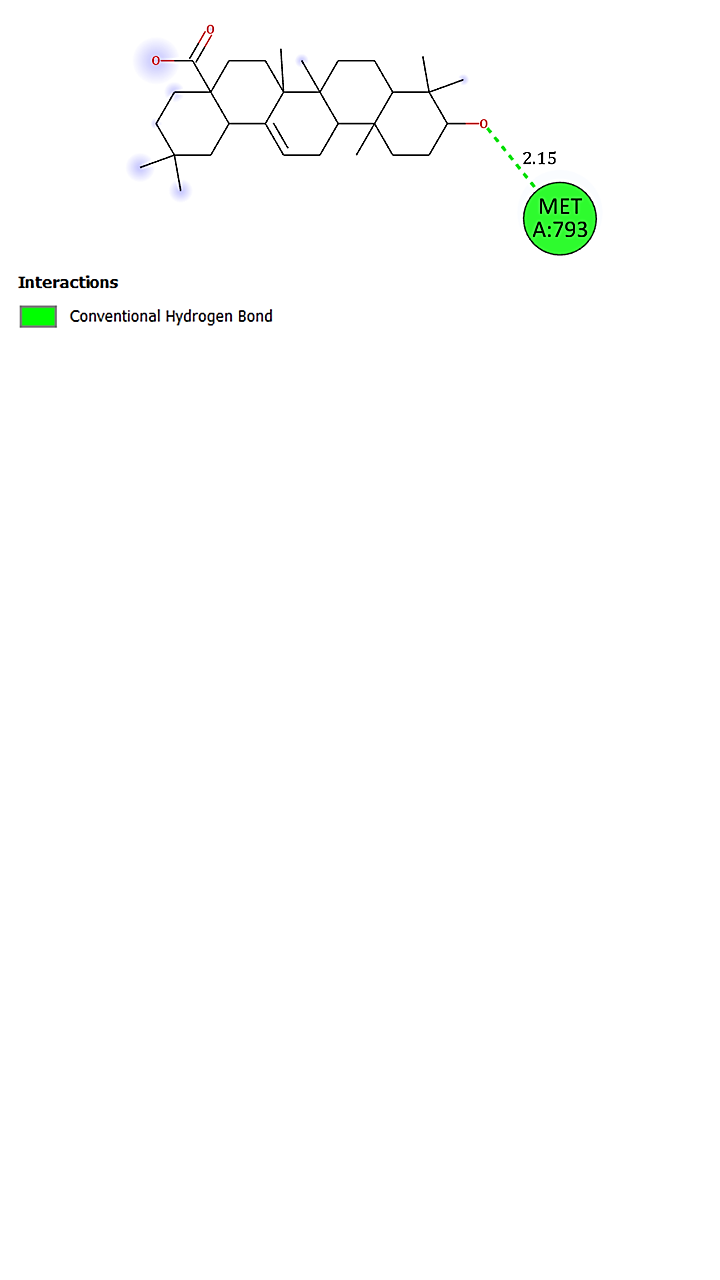** |
| **Lucidenic acid A** | |
| **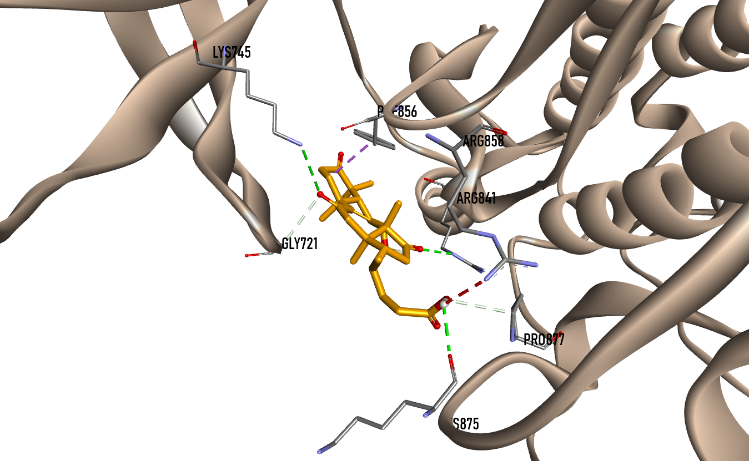** | **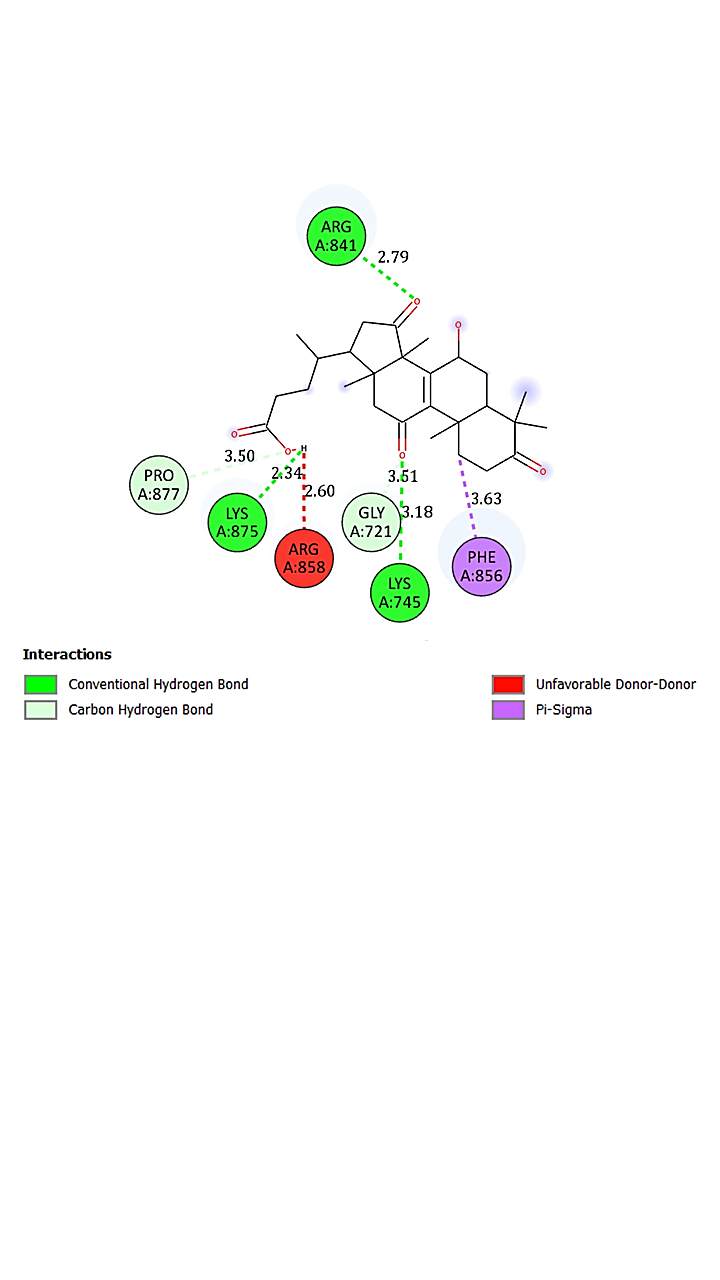** |
| **Hesperetin** | |
| **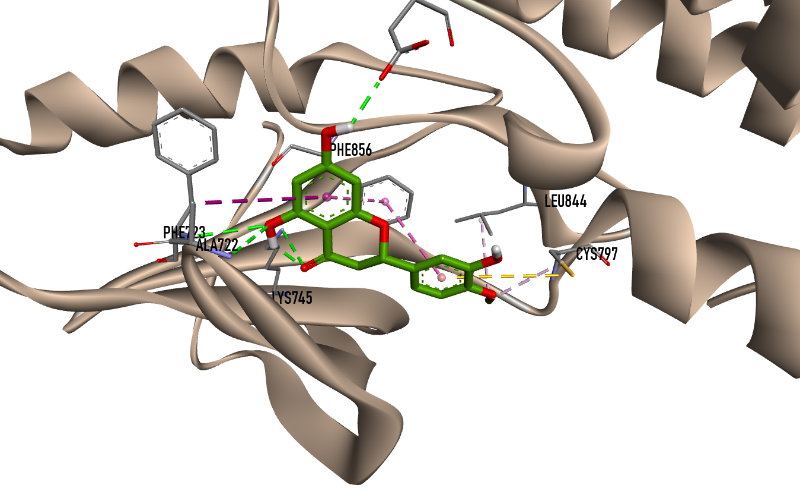** | **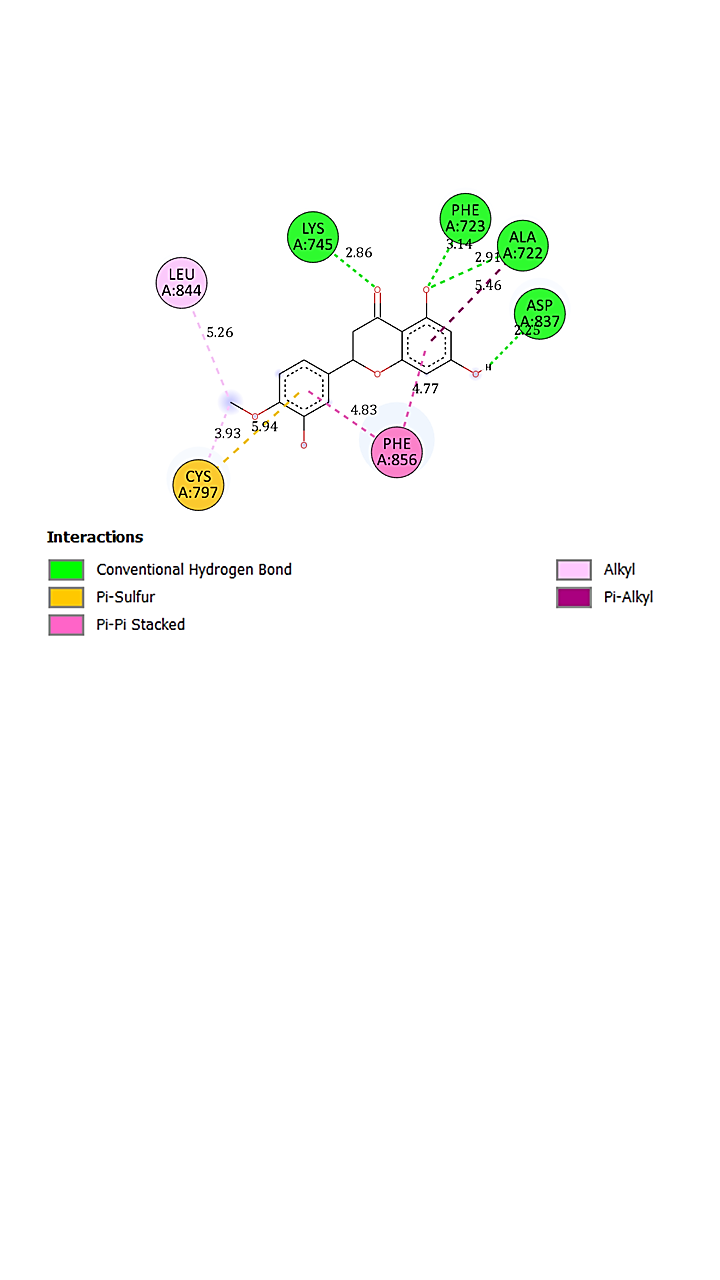** |
|  |  |
| **Acacetin** | |
| **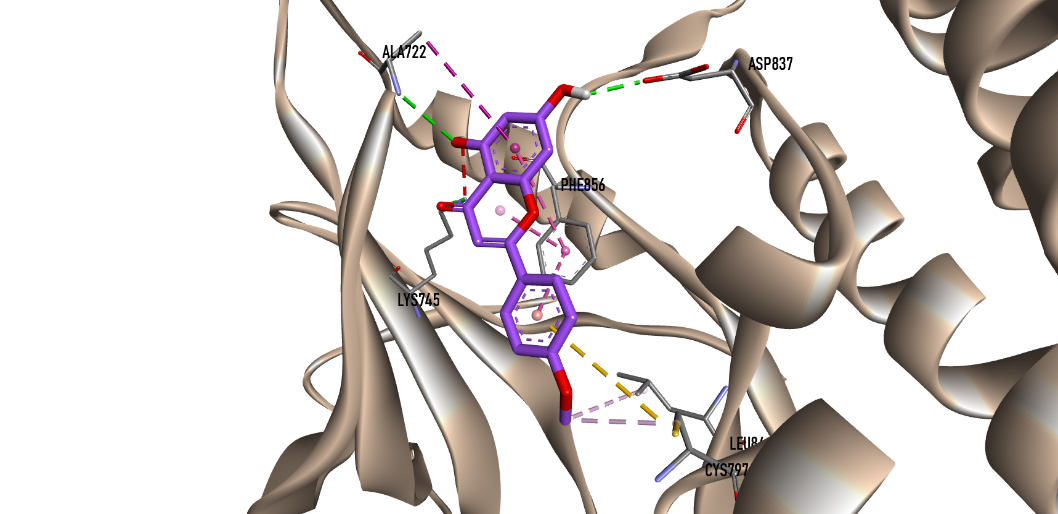** | **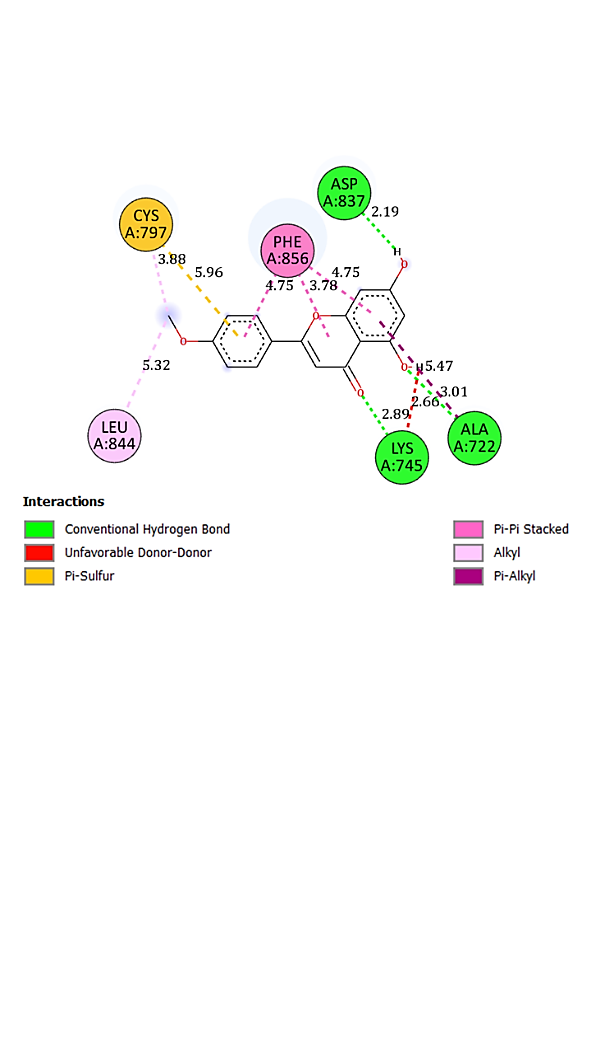** |
| **3,9-Dihydroeucomin** | |
| **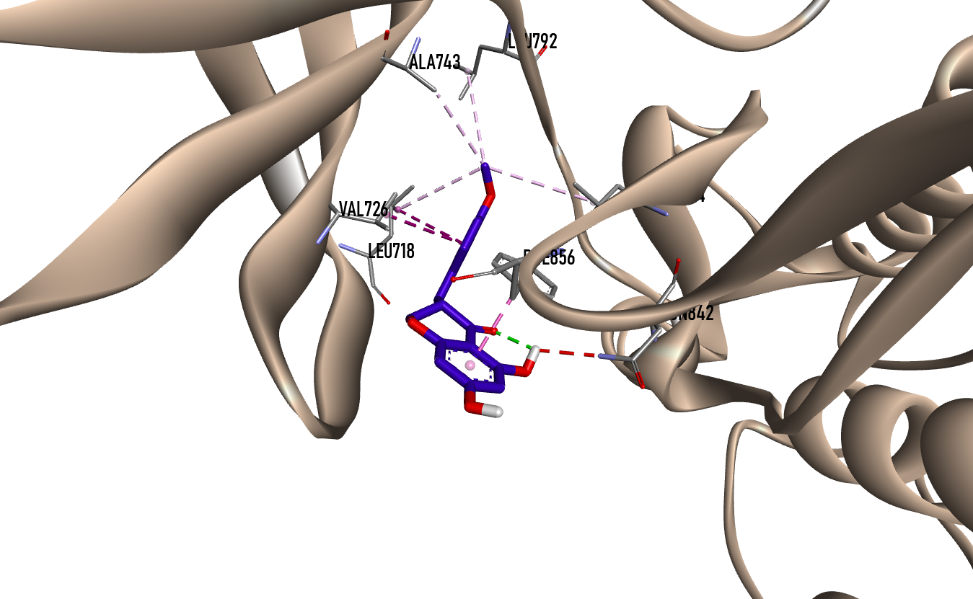** | **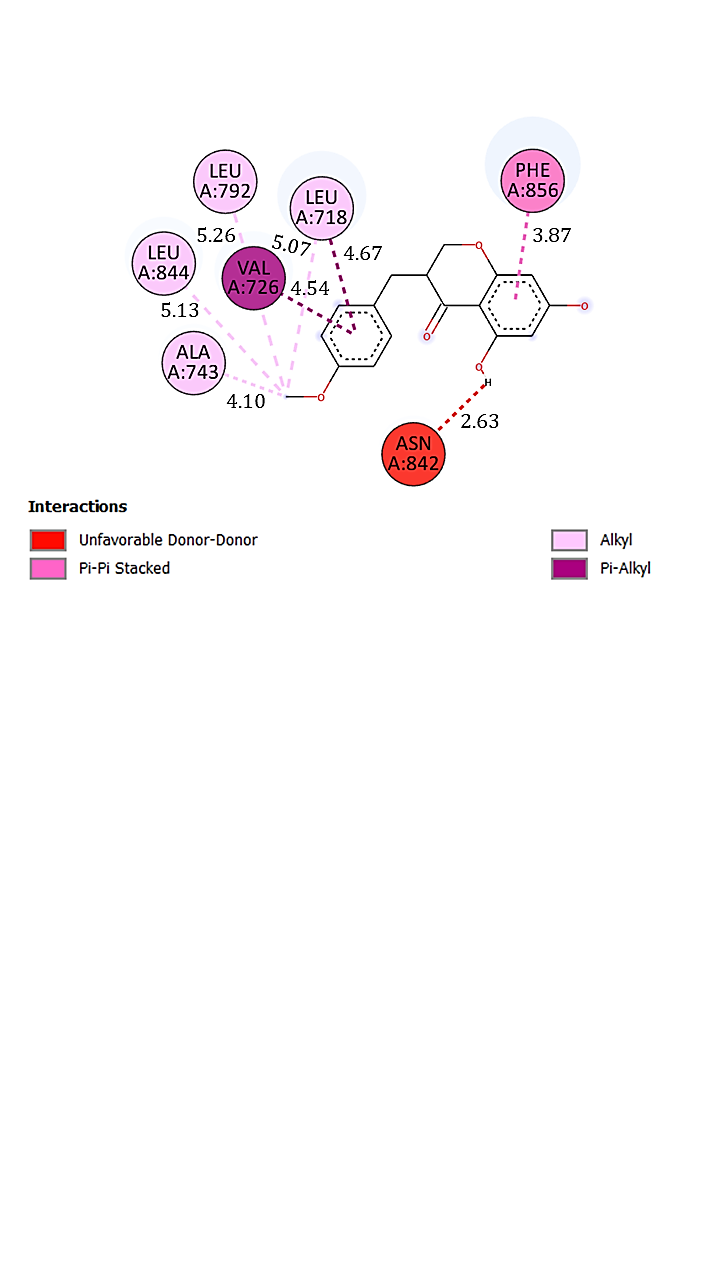** |

**Figure S4.** 3D and 2D representations of molecular interactions for the top five compounds exhibiting the strongest binding affinities to EGFR, arranged in descending order of binding strength.

| **Lucidenic acid A** | |
| --- | --- |
| **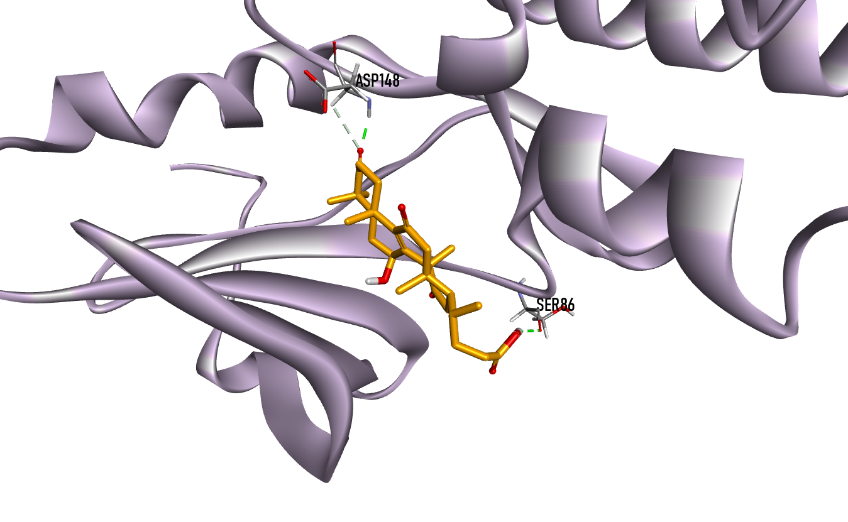** | **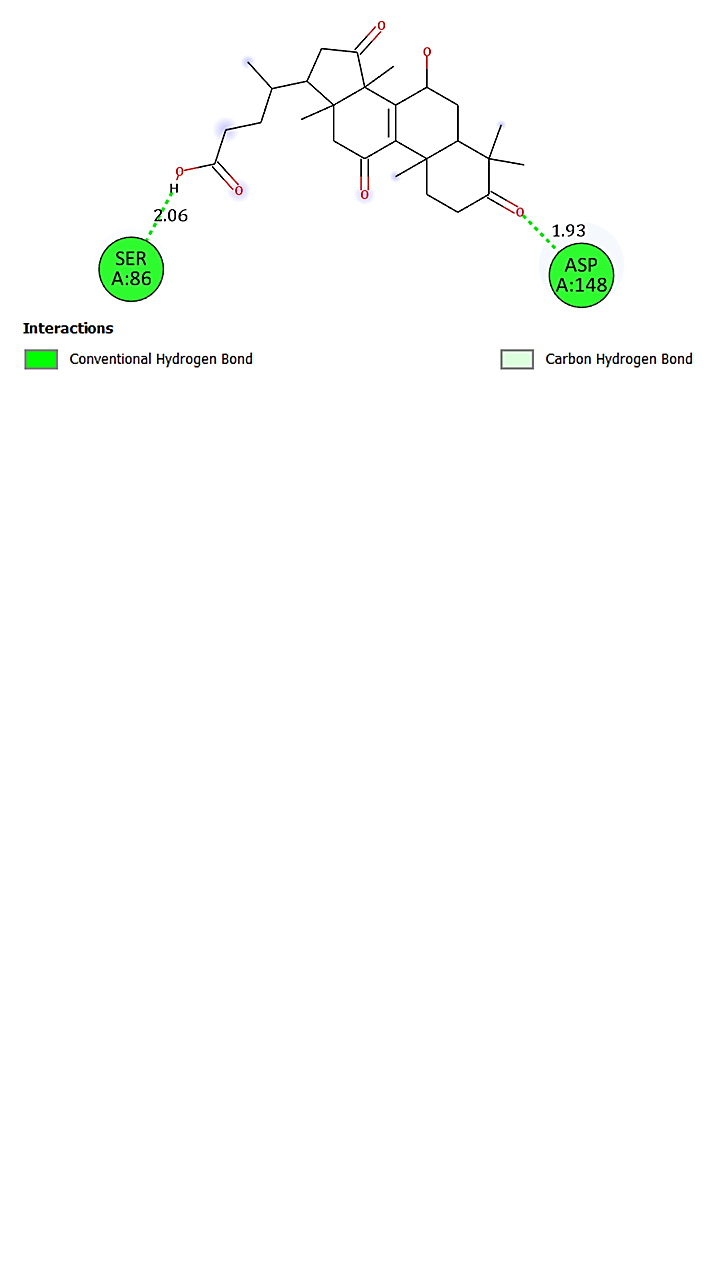** |
| **Oleanolic acid** | |
| **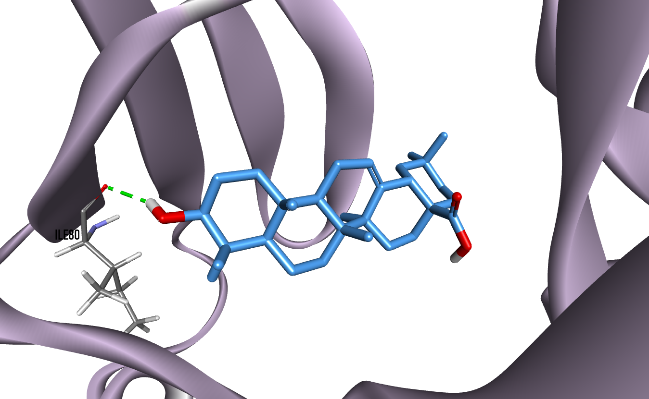** | **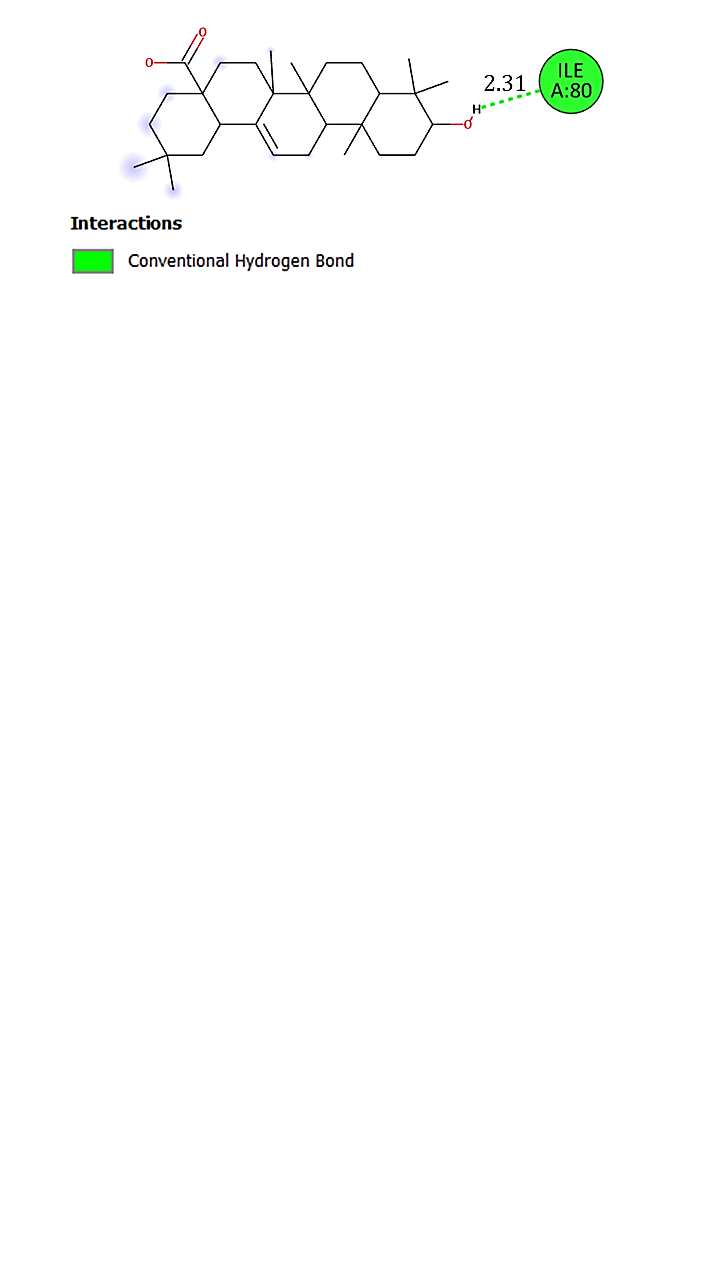** |
| **Pinocembrine** | |
| **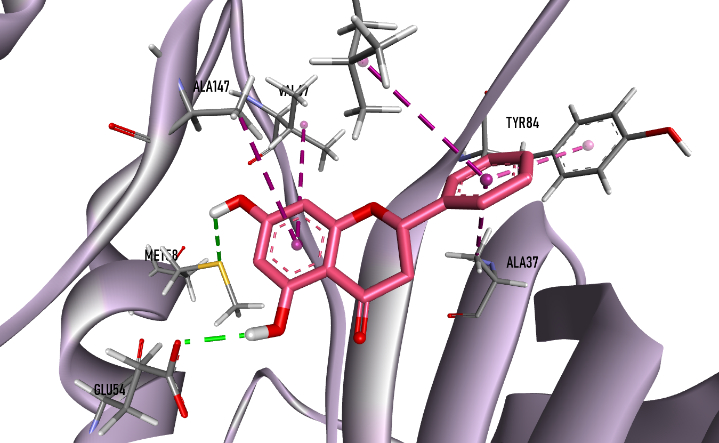** | **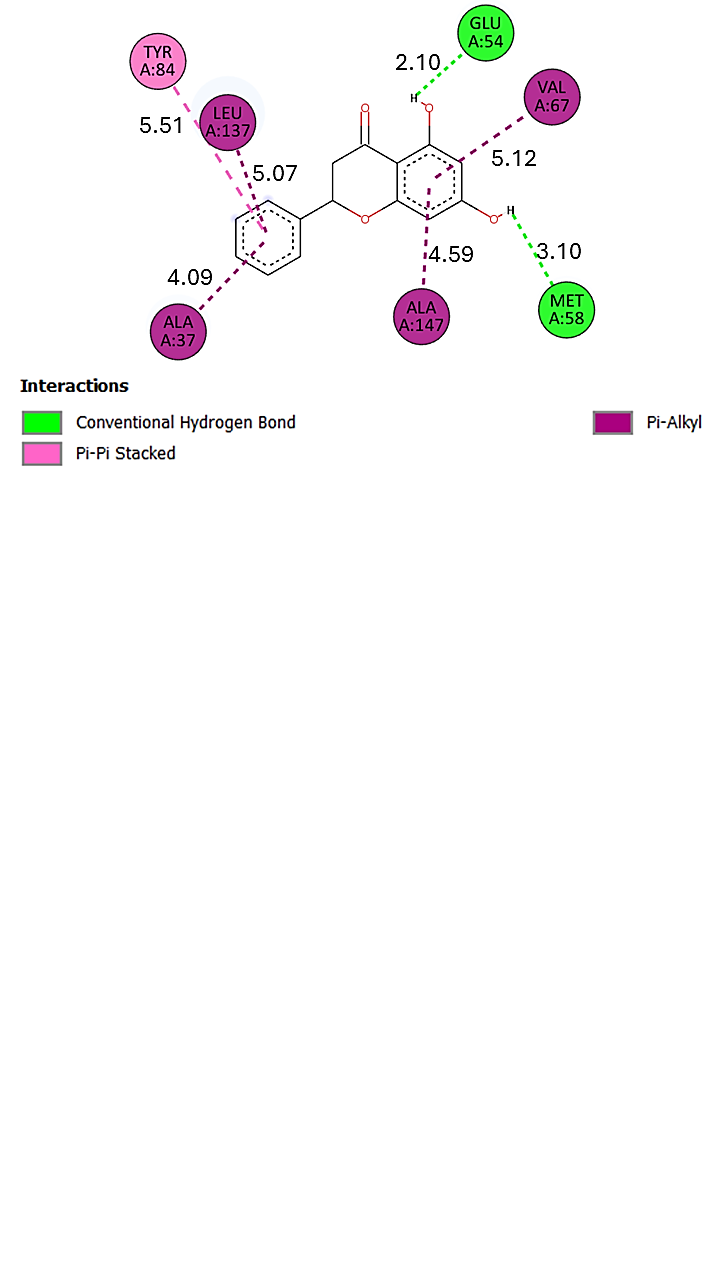** |
| **Acacetin** | |
| **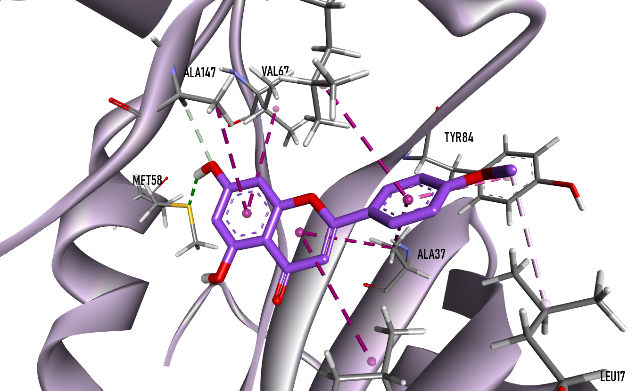** | **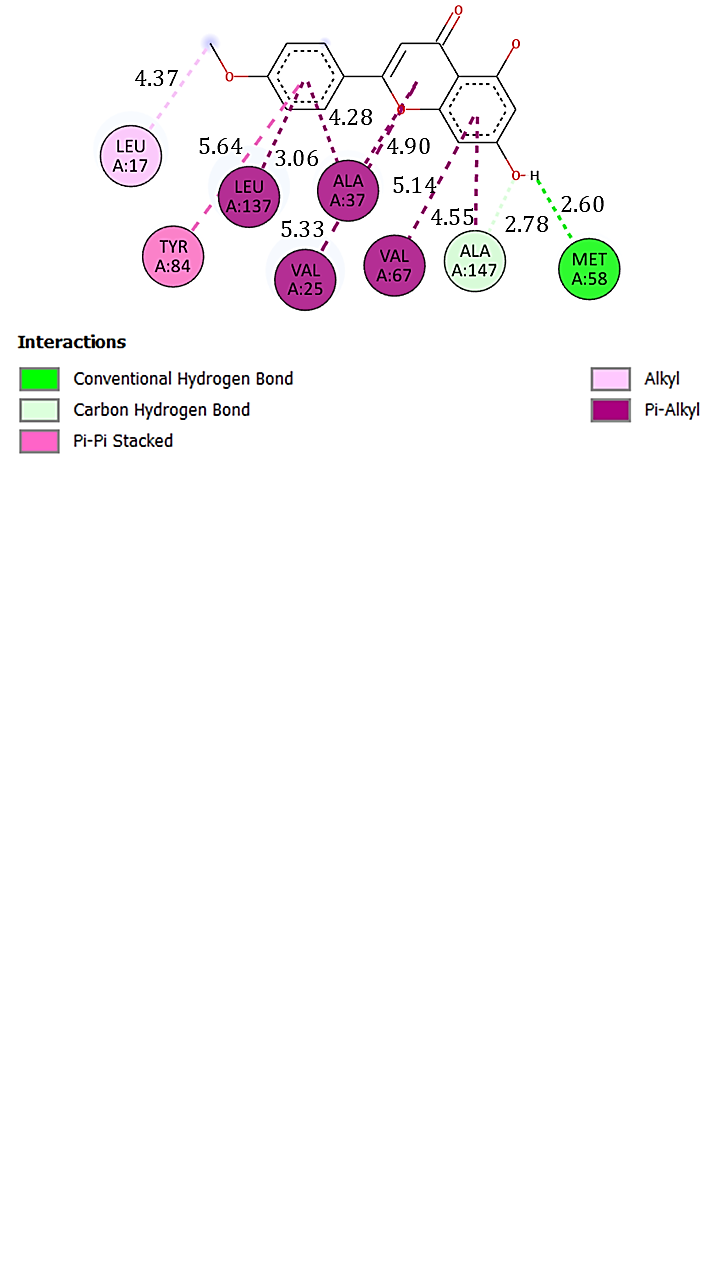** |
|  |  |
| **Hesperetin** | |
| **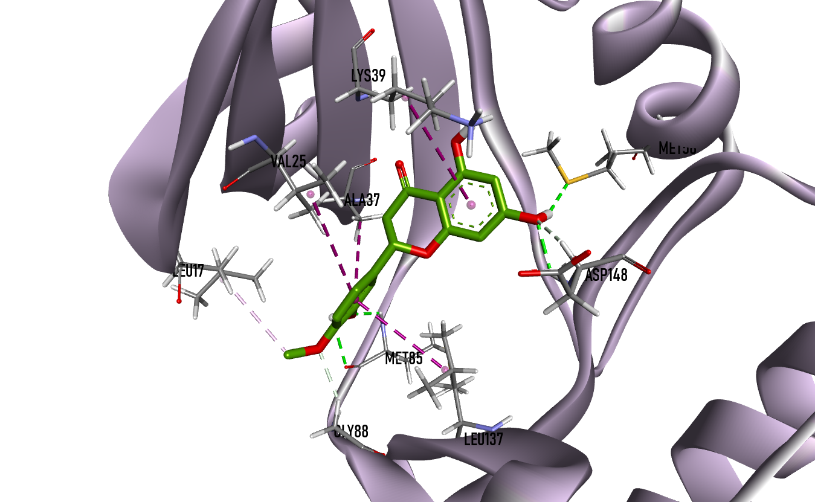** | **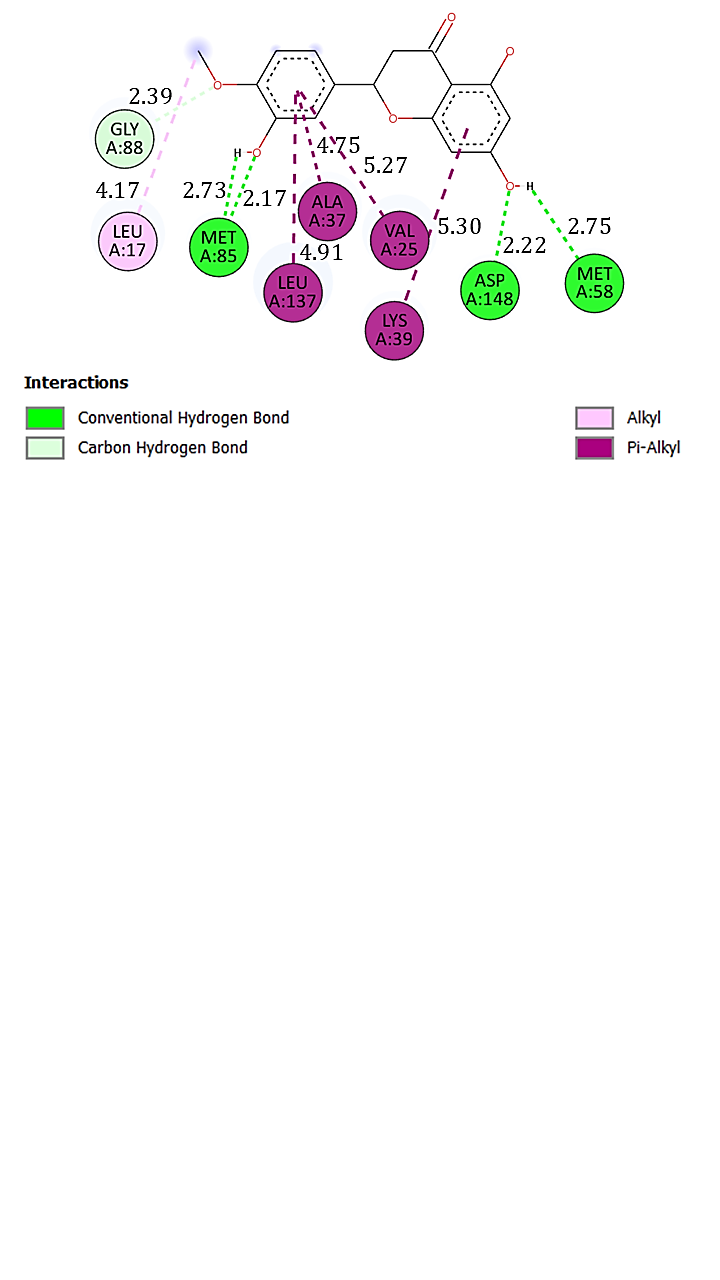** |

**Figure S5.** 3D and 2D representations of molecular interactions for the top five compounds exhibiting the strongest binding affinities to SRC, arranged in descending order of binding strength.

| **Hesperetin** | |
| --- | --- |
| **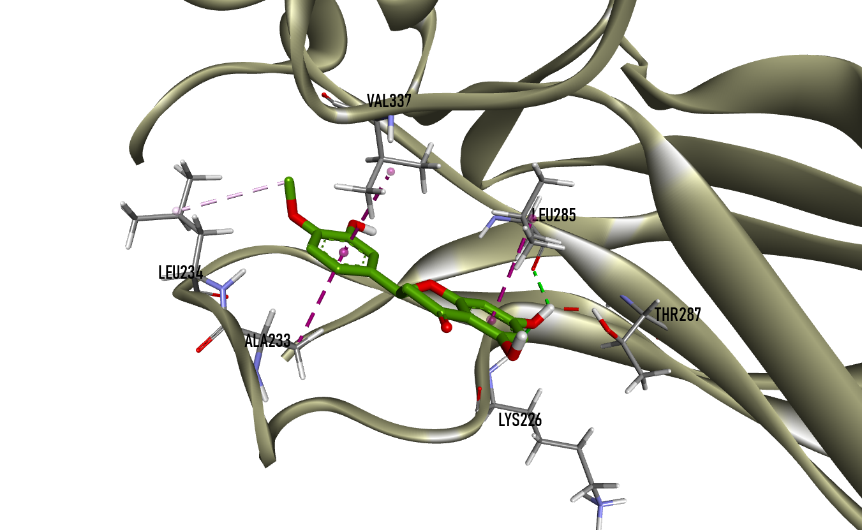** | **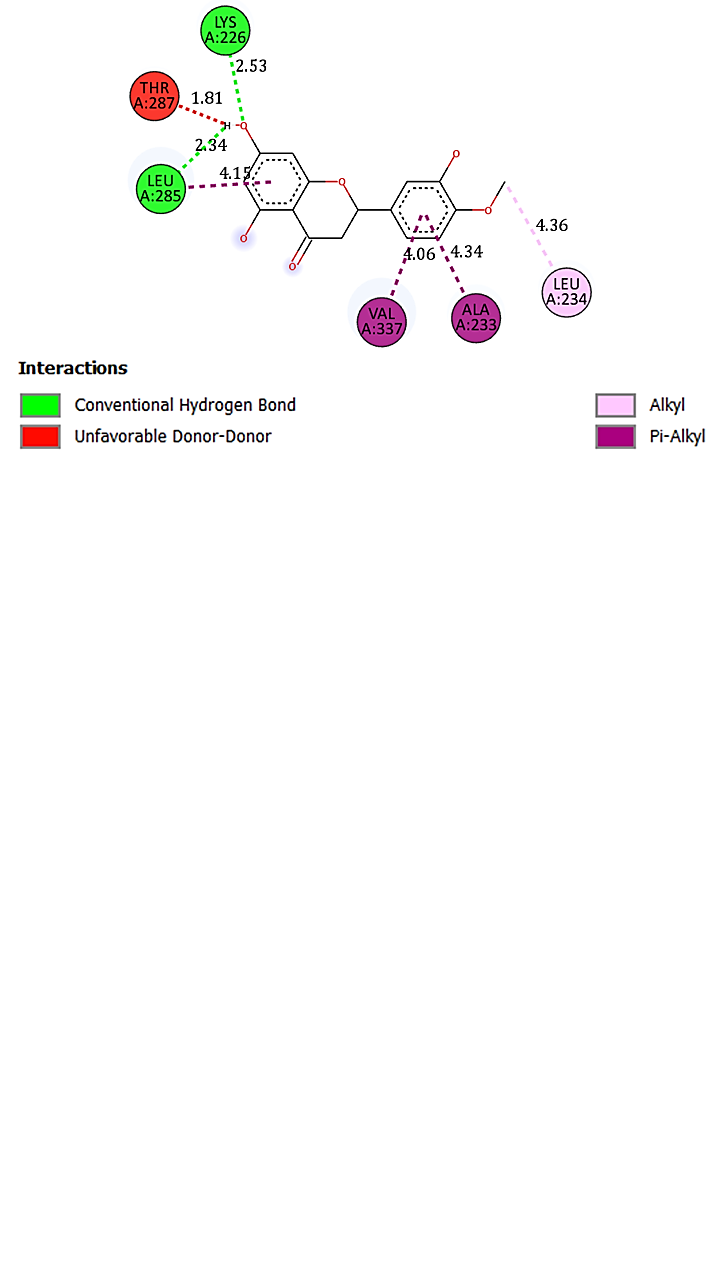** |
| **Pinocembrine** | |
| **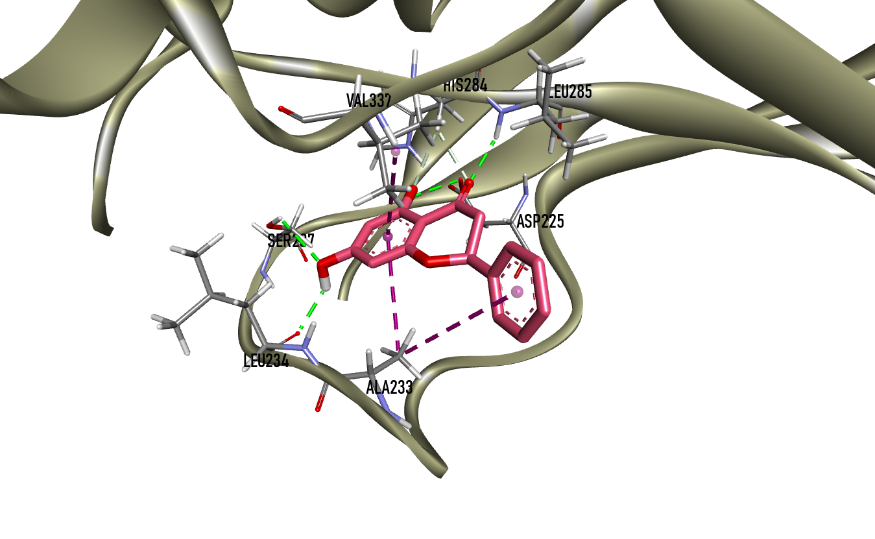** | **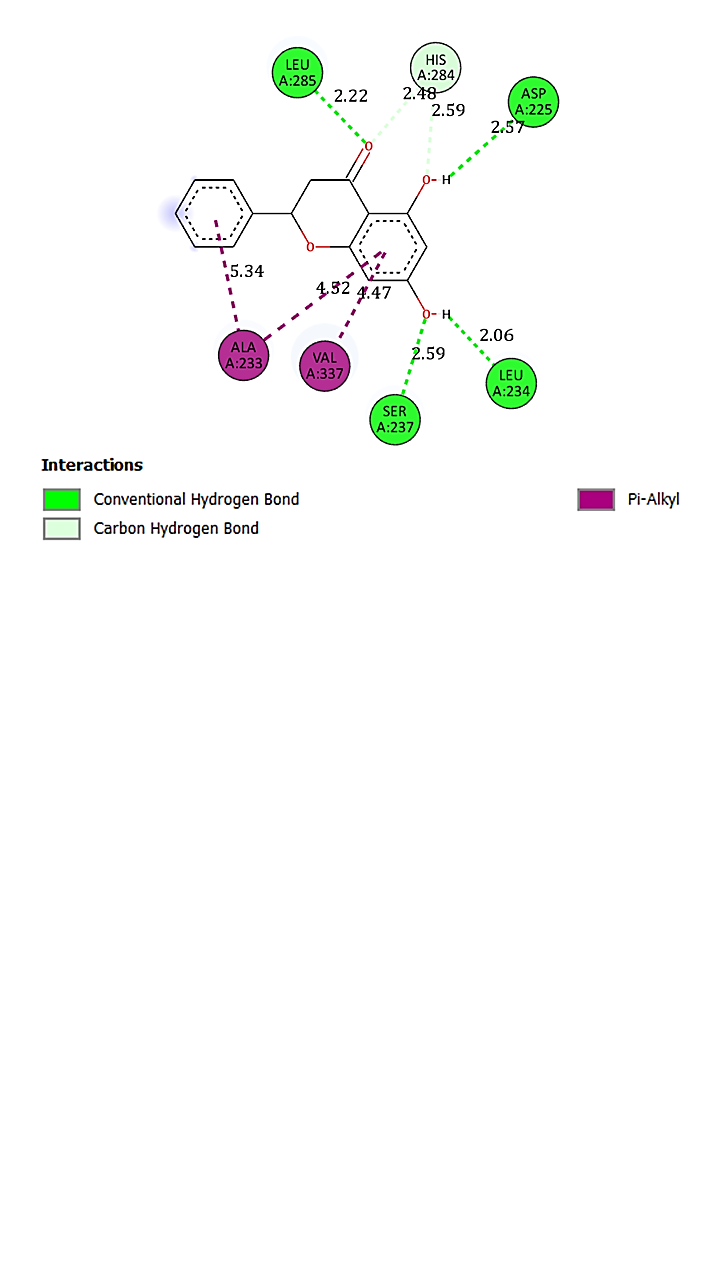** |
| **Acacetin** | |
| **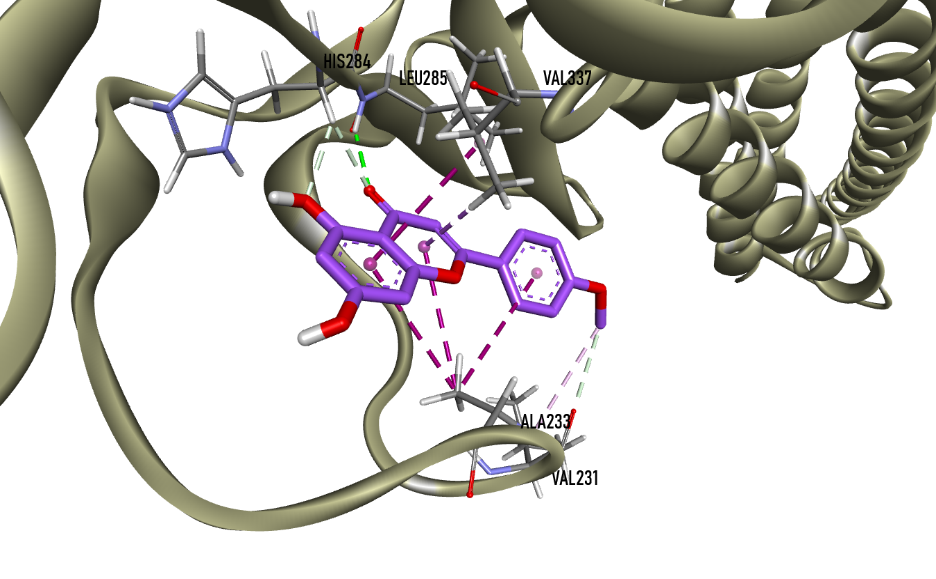** | **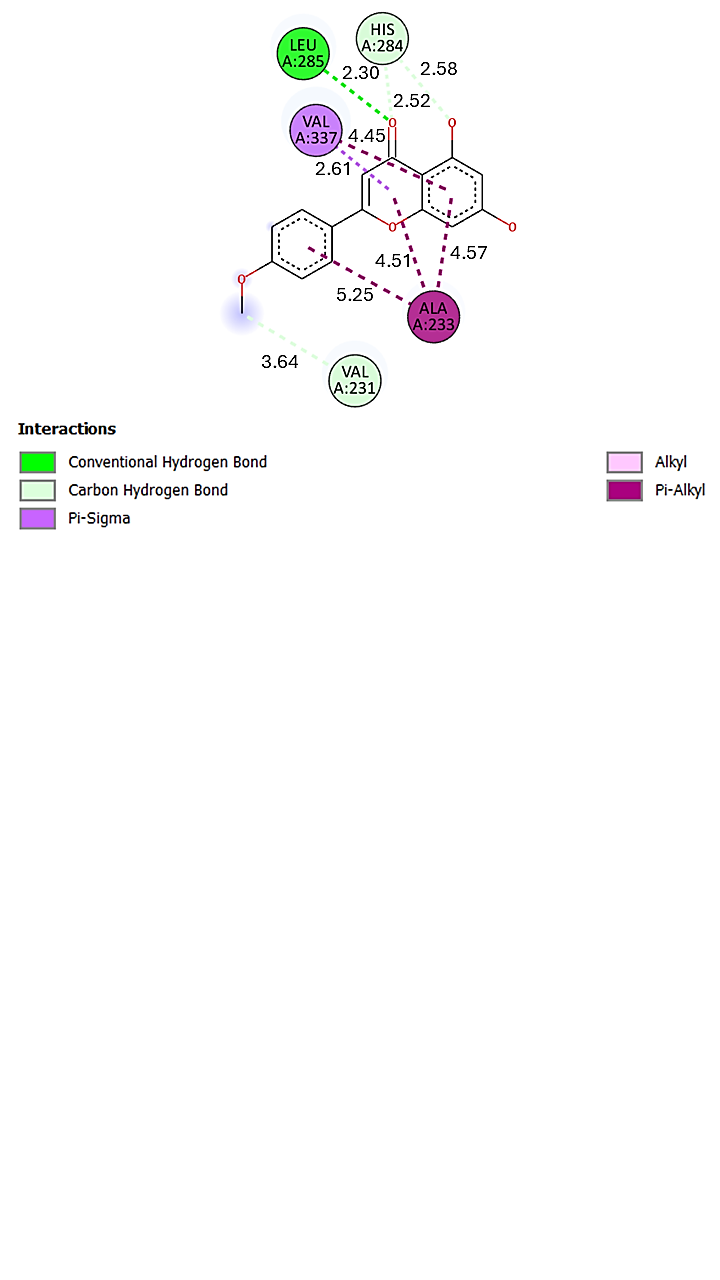** |
|  |  |
| **Kaempferol** | |
| **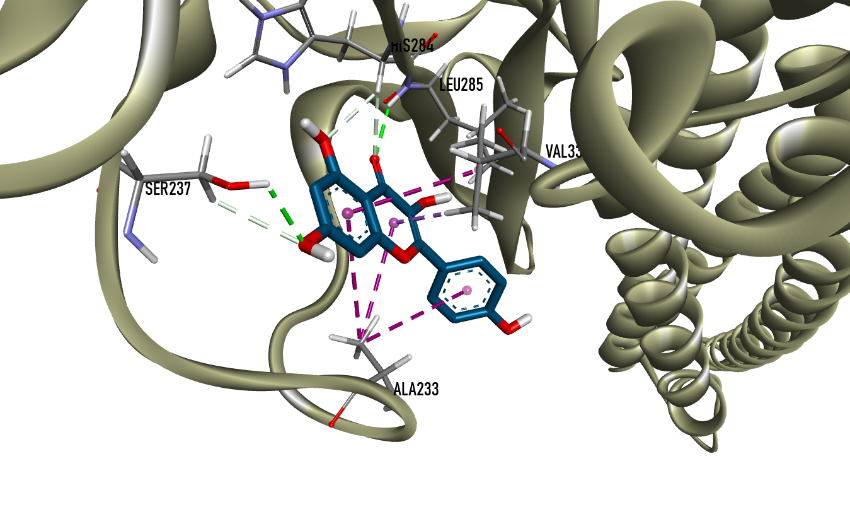** | **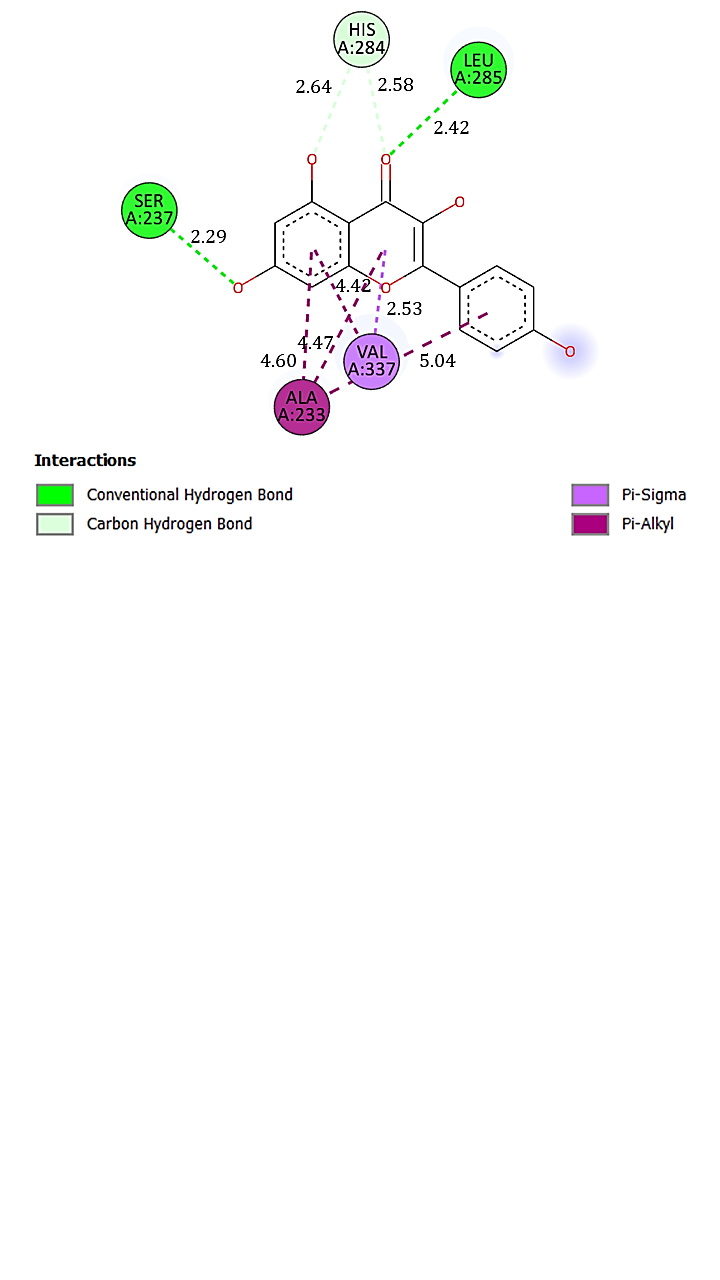** |
| **Pechueloic acid** | |
| **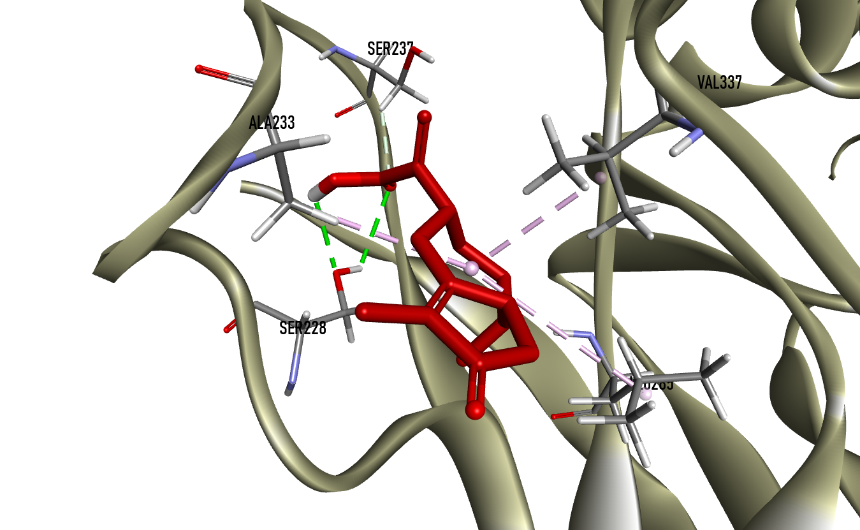** | **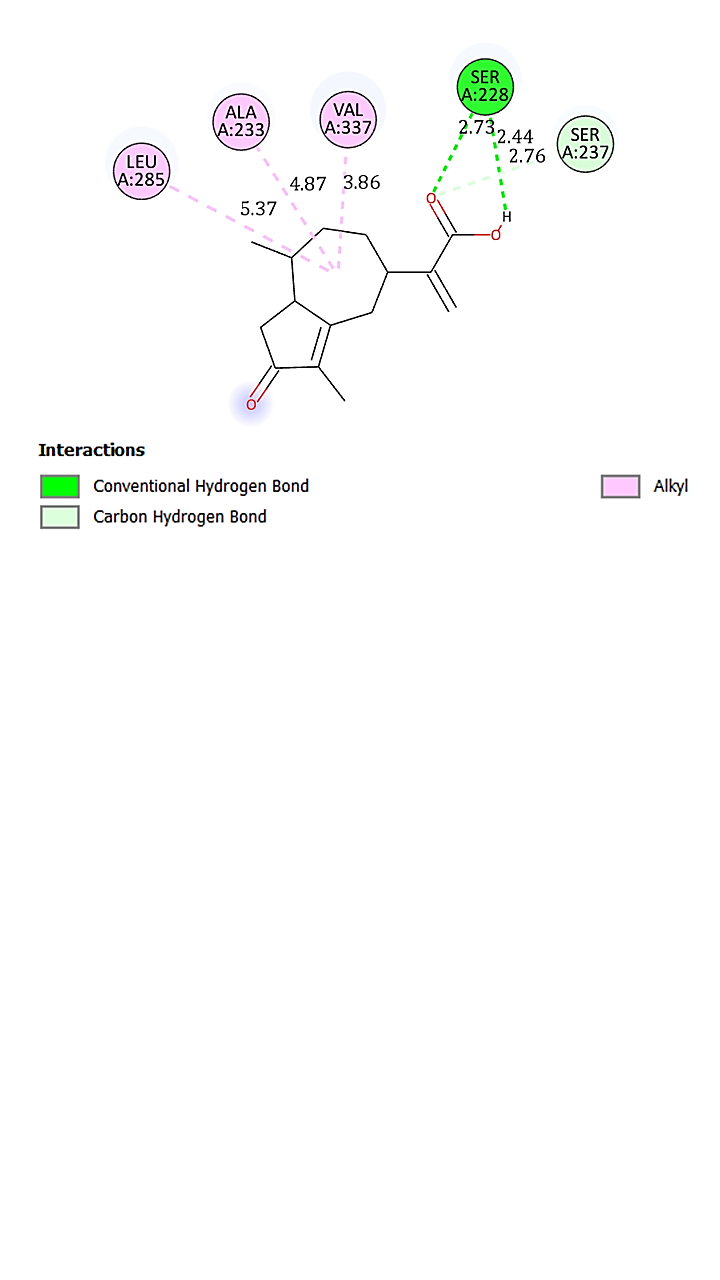** |

**Figure S6.** 3D and 2D representations of molecular interactions for the top five compounds exhibiting the strongest binding affinities to STAT3, arranged in descending order of binding strength.

| **Lucidenic acid A** | |
| --- | --- |
| **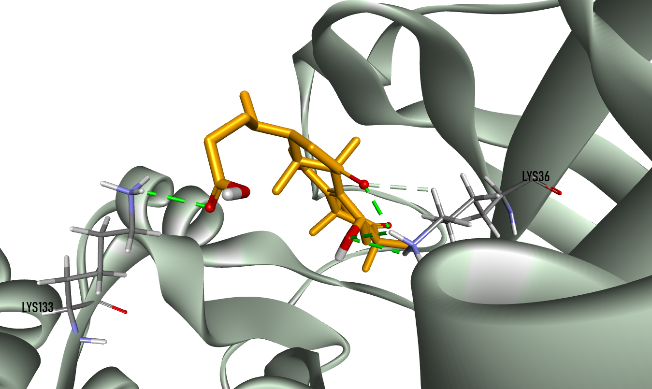** | **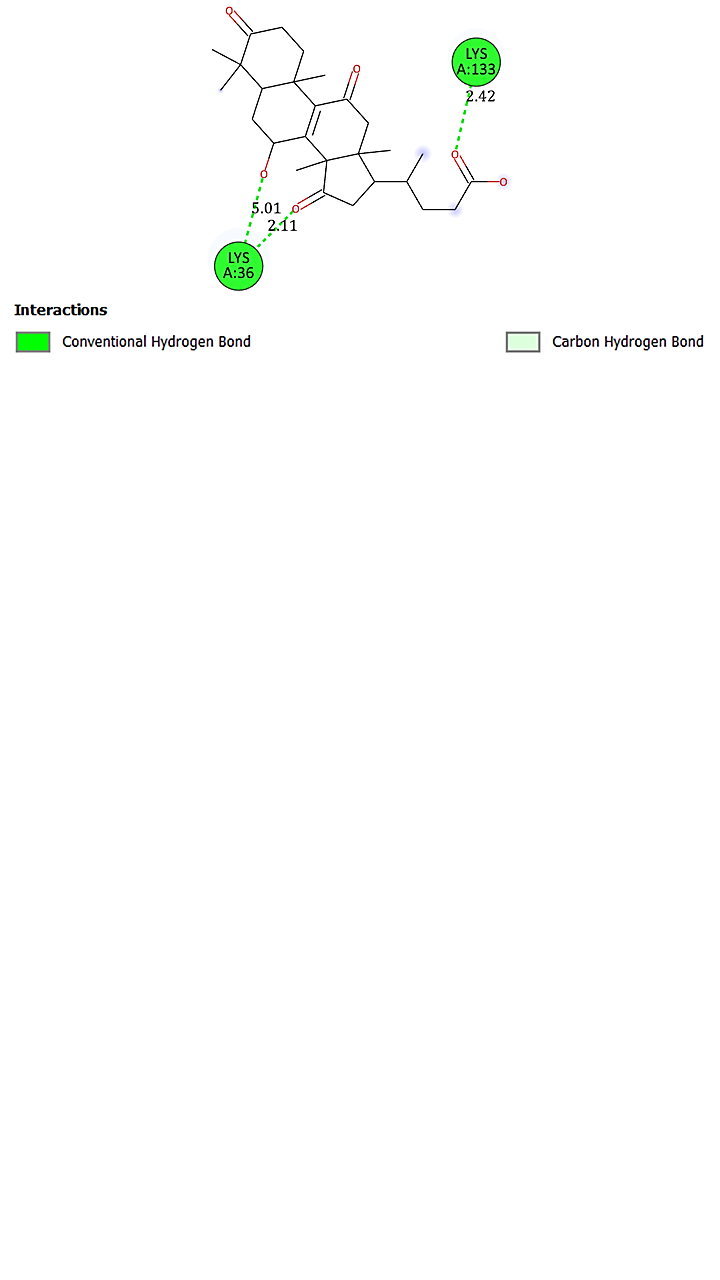** |
| **Hesperetin** | |
| **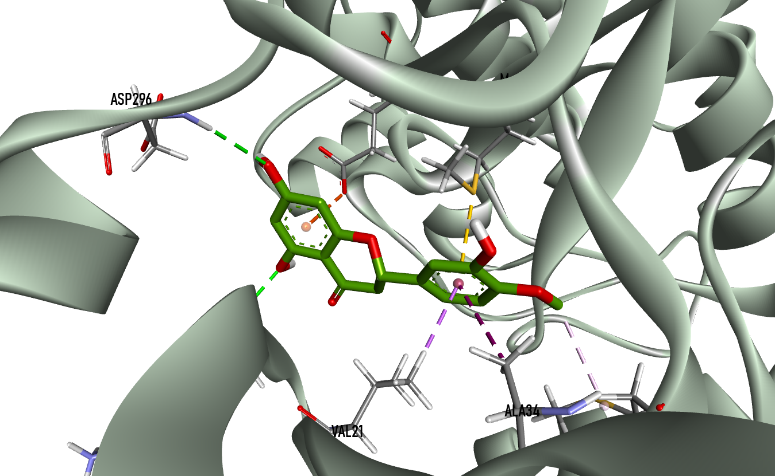** | **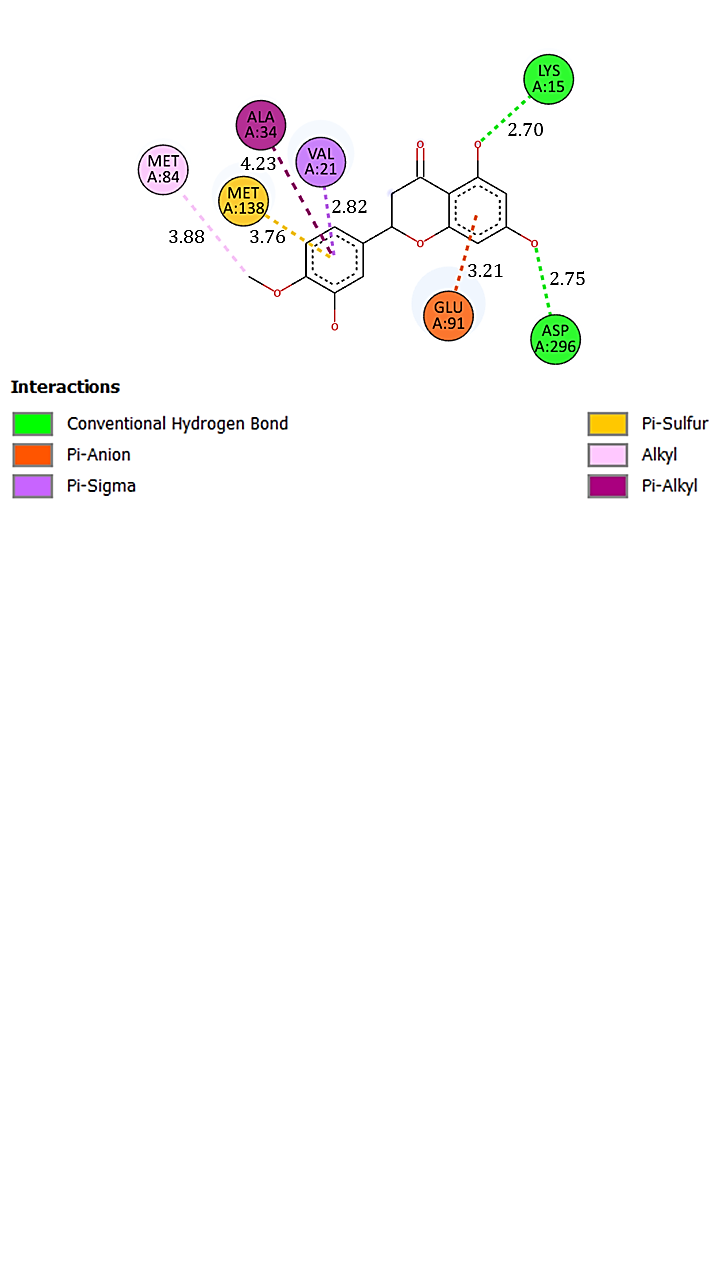** |
| **Pinocembrine** | |
| **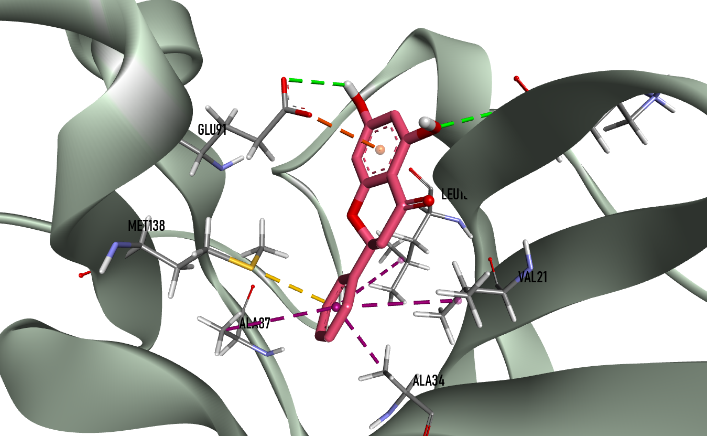** | **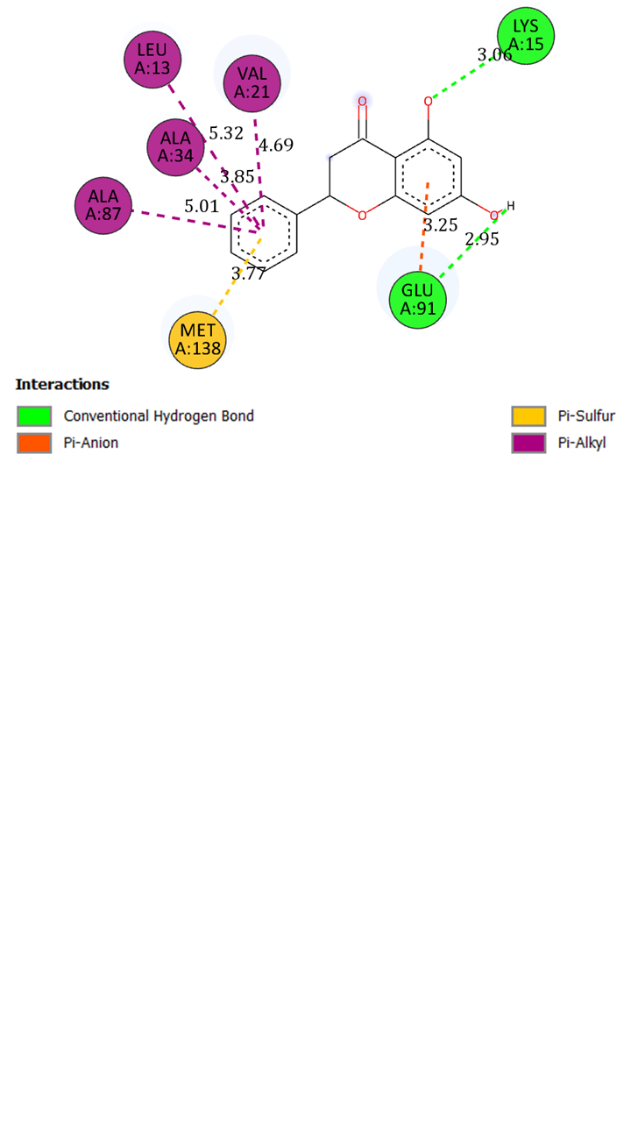** |
| **Acacetin** | |
| **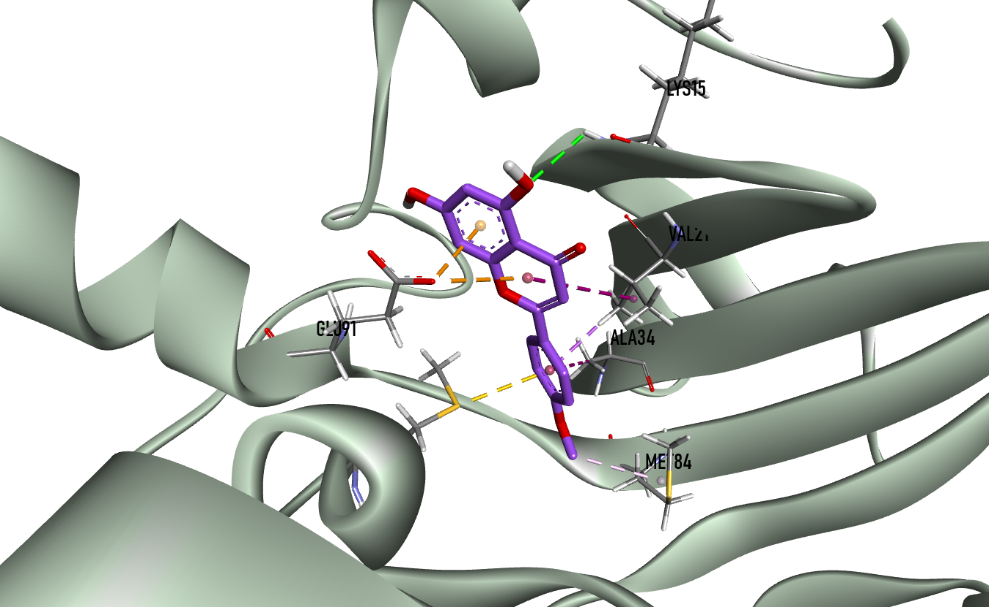** | **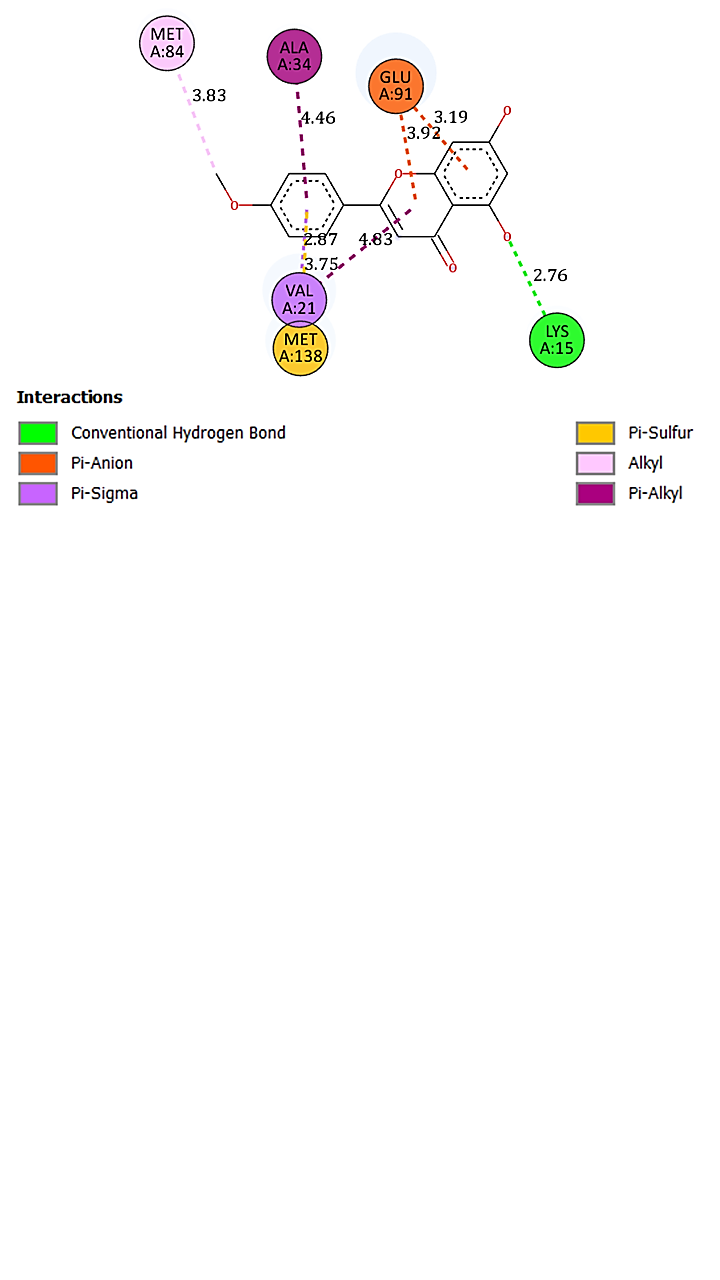** |
|  |  |
| **3,9-Dihydroeucomin** | |
| **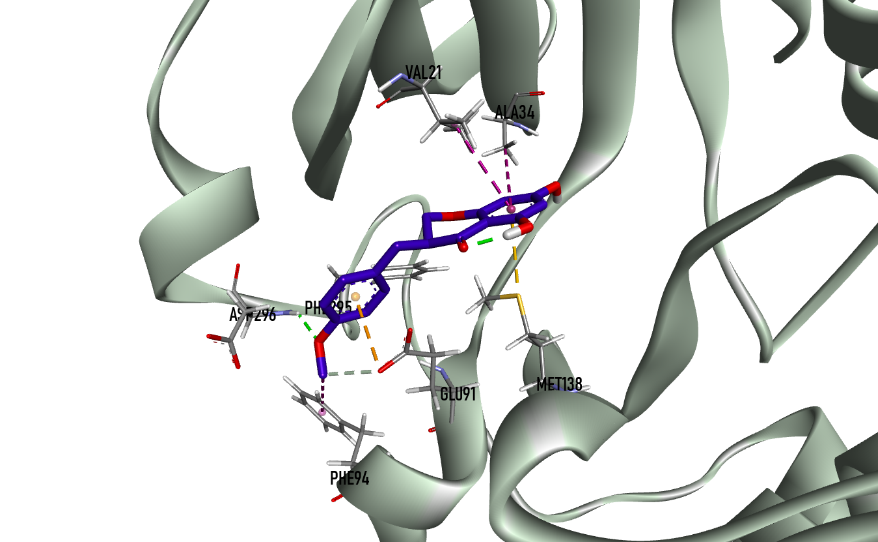** | **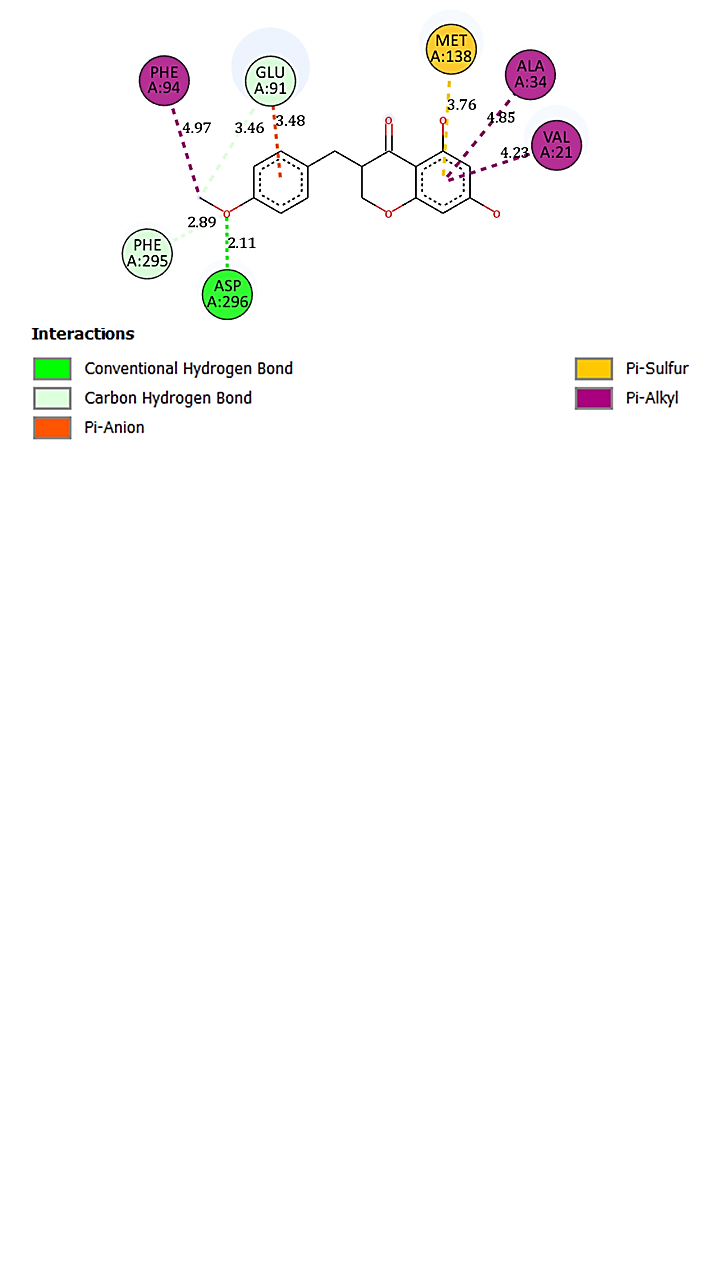** |

**Figure S7.** 3D and 2D representations of molecular interactions for the top five compounds exhibiting the strongest binding affinities to AKT1, arranged in descending order of binding strength.
