# Supplementary material for: Therapeutic Potential of Beaucarnea recurvata Leaf Extract Against Ulcerative Colitis: Integrating Phytochemical Profiling, Network Pharmacology, and Experimental Validation
Source: Int J Mol Sci. 2025 Dec 15;26(24):12053. doi: 10.3390/ijms262412053 (PMC12733345; doi:10.3390/ijms262412053)
Supplement: Supplementary file 1 [file ijms-26-12053-s001.zip › Supplementary Results_Figures S8-S12.docx]

**Supplementary Results (Figures S8-S12).**

Histopathological Analysis of Colonic Tissue Specimens

Comprehensive microscopic evaluation of H&E-stained colonic cross-sections revealed pronounced structural variations among experimental cohorts, establishing clear morphological criteria for disease severity and therapeutic efficacy assessment.

Control specimens displayed preserved histoarchitectural organization across all tissue compartments including mucosal, submucosal, muscular, and serosal layers (Figure S8). The mucosal compartment demonstrated characteristic stratification comprising epithelial surface, lamina propria, and underlying smooth muscle. Colonic crypts maintained uniform spatial arrangement extending perpendicularly from the luminal interface to the muscularis mucosae.

The surface epithelium consisted of mature columnar cells interspersed with mucin-producing goblet cells. The lamina propria contained loose connective tissue matrix with physiological densities of resident inflammatory cells.


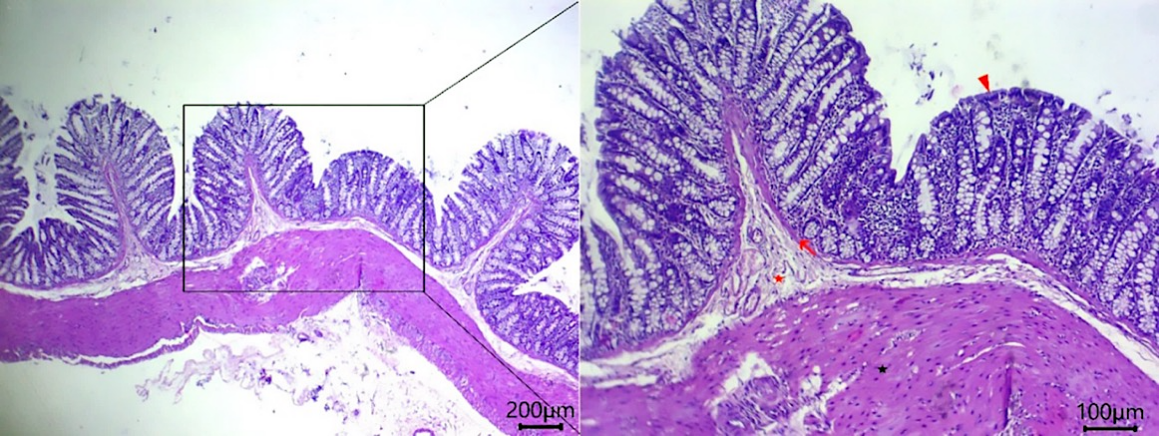


**Figure S8.** Representative photomicrographs of H&E-stained colonic sections from the control group, illustrating normal histological architecture. Key features include the lamina epithelialis (arrowhead), lamina propria and muscularis mucosa (arrow), submucosa (red star), muscular layer (black star), and serosa. Scale bars: 200 μm and 100 μm.

Colonic sections from the untreated diseased group exhibited severe active inflammation representing fulminant colitis with complete mucosal replacement by necrotic debris, fibrin networks, and fragmented crypt remnants (Figure S9 A-E). This case exemplifies the most severe degree of colitis with extensive transmural involvement and life-threatening complications. Bacterial colonization was evident as densely basophilic deposits overlying necrotic material. The submucosal compartment contained dense neutrophilic infiltrates, hemorrhagic extravasation, and fibrin deposition. Vessel wall necrosis with intensive neutrophilic infiltration characterized the submucosal vasculature. Muscular layers contained thrombosed vessels composed of fibrin, erythrocytes, and leukocytes adherent to the luminal surface. Inflammatory cells, granulation tissue, and fibrin networks replaced normal muscular architecture, while subserosal regions showed extensive erythrocyte extravasation. Additional sections from this case exhibited complete colonic wall replacement by necrotic material, fibrin threads, inflammatory infiltrates, fragmented crypts, and bacterial colonies (Figure S9 F-H). Heavy neutrophilic infiltration, bacterial overgrowth, fibrin deposition, and erythrocyte extravasation characterized the submucosal layer alongside vascular congestion and vessel wall hyalinization. Further sections revealed chronic active inflammation with remnants of necrotic surface epithelium adjacent to cystically dilated crypts (Figure S9 I-J). Dense infiltration of eosinophils, macrophages, lymphocytes, and fibrin threads with scattered neutrophils affected mucosal and submucosal compartments, accompanied by edema and vascular dilatation. The muscular layer contained necrotic myocytes with pyknotic nuclei and abundant inflammatory cell infiltration.

**
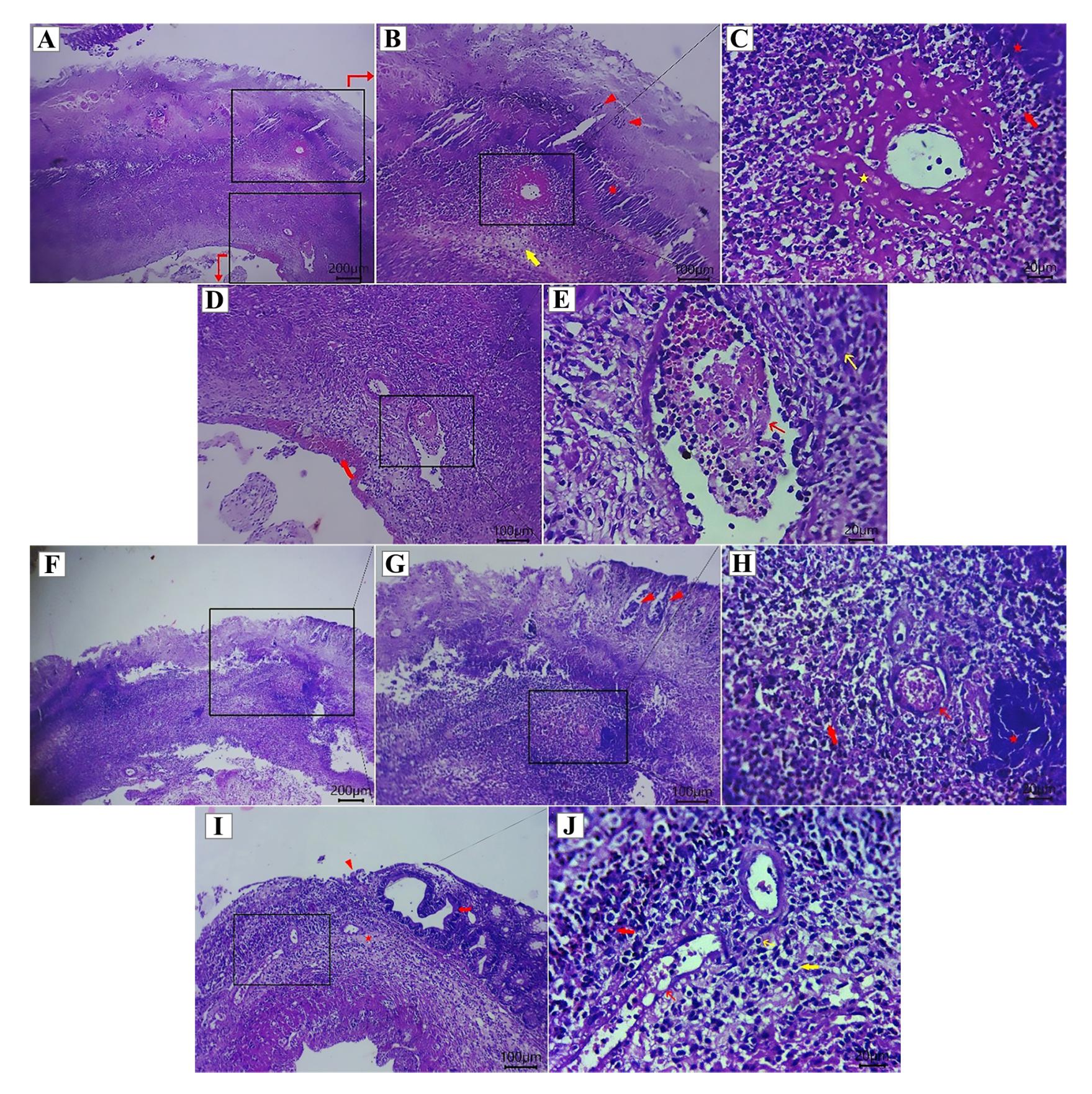
**

**Figure S9.** Representative H&E-stained colonic sections from the diseased group, showing histopathological features of chronic active and advanced ulcerative colitis. (A–B) Mucosal surface replaced by necrotic debris, fibrin, and crypt remnants (red arrowheads), with diffuse bacterial colonies (red stars), marked neutrophilic infiltration, submucosal hemorrhage, and fibrin deposition (thick yellow arrows). (C) Fibrinoid necrosis in submucosal vessels (yellow star) with bacterial colonies (red star), and perivascular neutrophilic infiltration (thick red arrows). (D–E) Mononuclear infiltrates (thin yellow arrows), granulation tissue, and fibrin encase thrombosed vessels (thin red arrows); subserosal erythrocyte extravasation (curved red arrow). (F–H) Colonic wall extensively necrotic with crypt remnants (arrowheads), dense neutrophilic infiltrates (thick red arrow), bacterial colonies (red star), fibrin, erythrocyte extravasation, congested and hyalinized vessels (thin red arrow). (I–J) Necrotic surface epithelium (red arrowhead), dilated crypts (curved red arrow), mucosal and submucosal infiltration by eosinophils (thick red arrow), macrophages (thick yellow arrow), lymphocytes (thin yellow arrow), beside perivascular edema (red star), and dilated vasculature (thin red arrow); muscular layer shows necrotic myocytes with pyknotic nuclei. Scale bars: 200 μm, 100 μm, and 20 μm.

BRLE administration produced dose-related morphological improvements with distinct patterns of tissue restoration. Examined sections from colon of rats at the BRLE100 group showed mucoid exudate adherent to colonic epithelium with persistent mucosal ulceration and exposed muscularis toward the luminal surface (Figure S10 A-B). Hyalinized or necrotic muscular tissue was evident alongside fibrin deposition and scattered inflammatory cells in the submucosal layer. Serosal exudation was also observed. Additional sections exhibited focal chronic active inflammation with mucosal replacement by distorted epithelial remnants, abundant neutrophils, lymphocytes, macrophages, edematous fluid, and fibrin (Figure S10 C-E). Hyperplastic or regenerative crypts were present adjacent to ulcerated regions, with submucosal edema persisting. Further sections demonstrated ulcerated mucosal surfaces with dense inflammatory infiltration primarily comprising neutrophils, lymphocytes, edematous fluid, and erythrocytes (Figure S10 F). Architectural distortions included cryptitis with neutrophilic luminal infiltration, crypt atrophy, dilatation, branching, irregular configuration, and metaplastic changes with increased goblet cell populations. The submucosal layer contained heavy inflammatory infiltration, predominantly neutrophils and lymphocytes, with persistent edematous fluid.


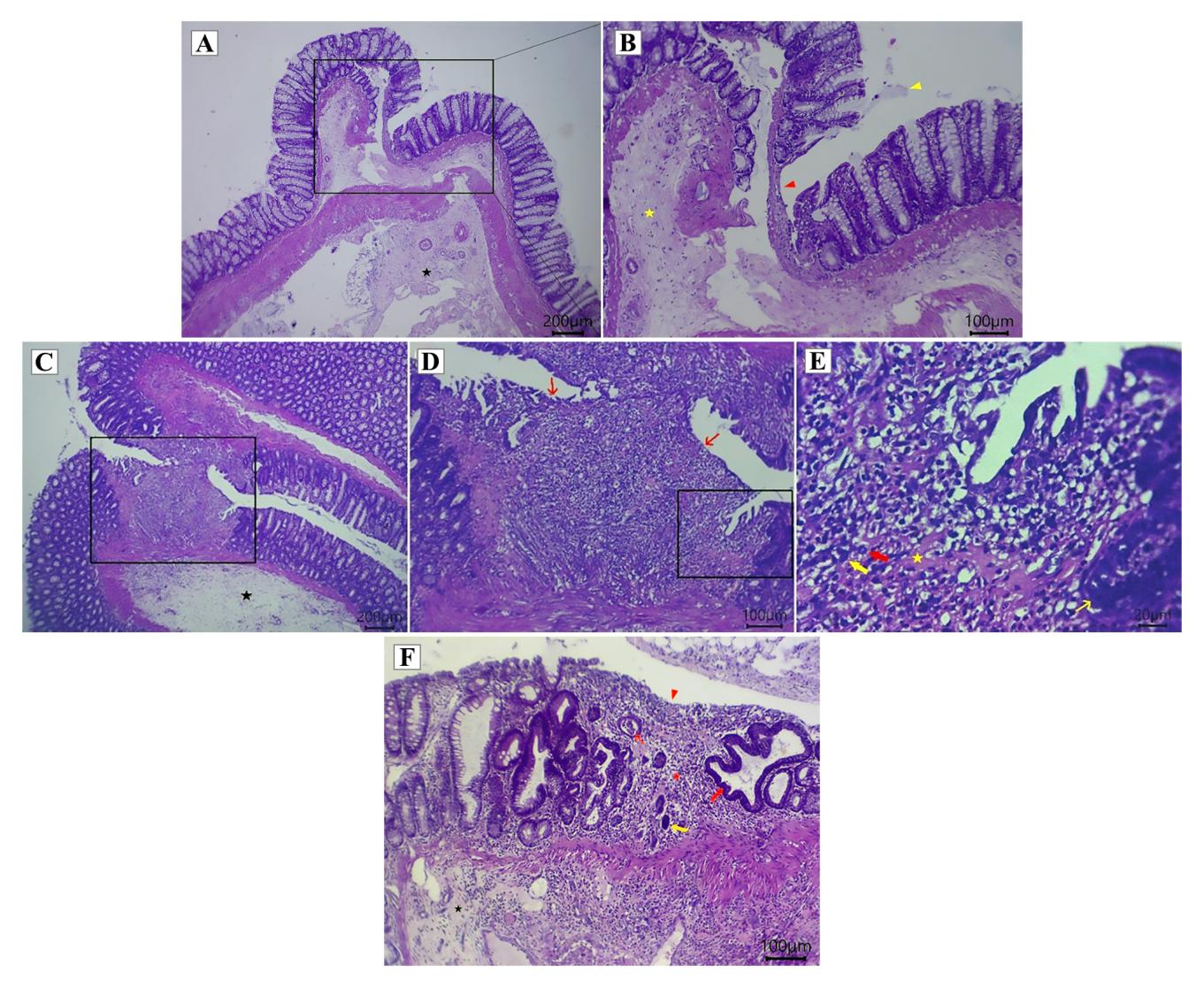


**Figure S10.** Representative H&E-stained colonic sections from the BRLE100-treated group (*Beaucarnea recurvata* leaf extract, 100 mg/kg/day), demonstrating features of chronic active ulcerative colitis. (A–B) Persistent mucosal ulceration with adherent mucous exudate (yellow arrowhead), protrusion and hyalinization/necrosis of the tunica muscularis (red arrowhead), adjacent fibrin deposits, sparse submucosal inflammation (yellow star), and serosal exudation (black star). (C–E) Distorted mucosal epithelium (thin red arrows) with abundant neutrophils (thick red arrow), macrophages (thick yellow arrow), lymphocytes, submucosal edema (black star), and fibrin (yellow star). Regenerating crypts (thin yellow arrow) are noted near ulcerated areas. (F) Ulcerated mucosa (red arrowhead) with dense inflammatory exudates (red star), cryptitis (thin red arrow), atrophied (curved yellow arrow) and distorted crypts (curved red arrow), metaplastic goblet cell changes, and marked submucosal edema (black star). Scale bars: 200 μm, 100 μm, 20 μm.

Examined sections from the BRLE200 group retained areas of chronic active inflammation with submucosal exposure at the luminal surface (Figure S11 A-C). Adjacent to ulcerated regions, dense inflammatory infiltration accompanied distorted crypts capped by hyperplastic or regenerative epithelium. Additional sections showed healed ulcerative areas characterized by regenerated surface epithelium and subepithelial granulation tissue with moderate inflammatory cell infiltration (Figure S11 D). Hyperplastic or regenerative crypts were present alongside cystically dilated structures. The submucosal layer displayed edema with reduced inflammatory cell density. Further sections exhibited healed ulcerative regions appearing as depressed surfaces relative to surrounding mucosa, with regenerated epithelium and crypts (Figure S11 E). Subepithelial areas were typically devoid of crypts and infiltrated with mononuclear inflammatory cells. The muscularis showed myolytic changes with inflammatory infiltration extending to the submucosal surface alongside edematous fluid.


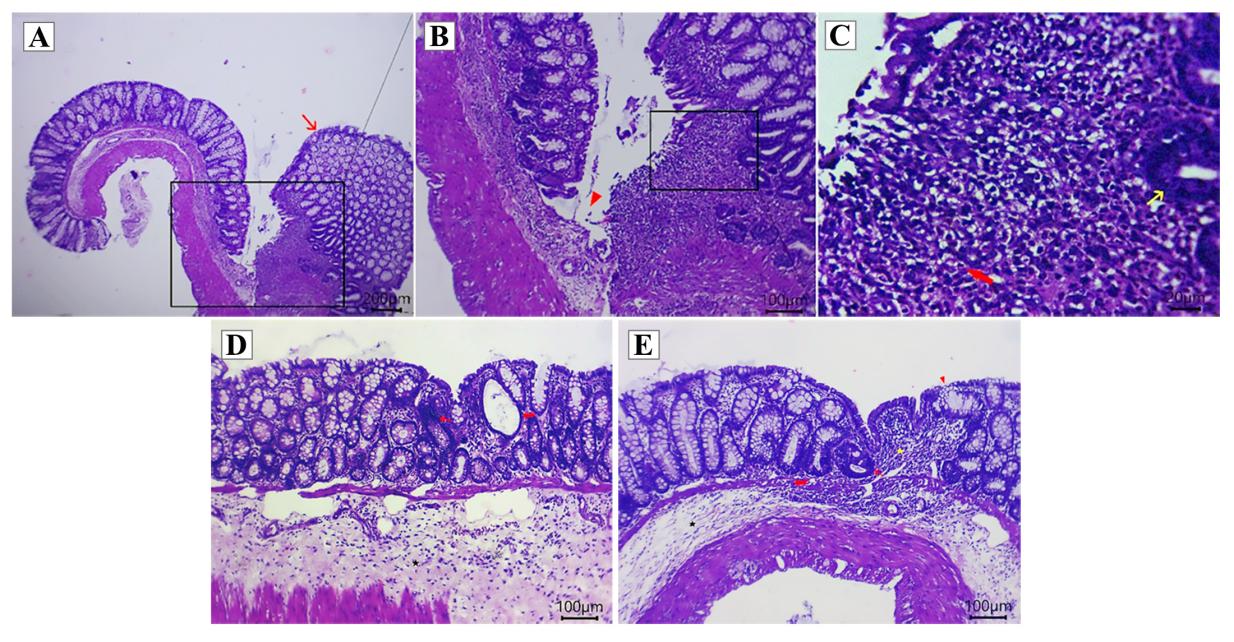


**Figure S11.** Representative H&E-stained colonic sections from the BRLE200-treated group (*Beaucarnea recurvata* leaf extract, 200 mg/kg/day), showing residual features of chronic active ulcerative colitis and evidence of mucosal regeneration. (A–C) Submucosal protrusion toward the lumen (red arrowhead), hyperplastic/regenerating epithelium (thin red arrow) and crypts (thin yellow arrow), with dense inflammatory infiltration (thick red arrow) and distorted crypt architecture. (D) Regenerating surface epithelium and crypts (thin red arrow), cystically dilated crypts (curved red arrow), and submucosal edema with minimal inflammation (black star). (E) Regenerated surface epithelium (red arrowhead), crypt (thin red arrow), loss replaced by mononuclear infiltrates (yellow star), myolysis of tunica muscularis (curved red arrow), and edematous submucosa (black star). Scale bars: 200 μm, 100 μm, 20 μm.

Examined sections from the BRLE400 group displayed persistent inflammation with ulcerated areas replaced by numerous inflammatory cells, primarily lymphocytes, macrophages, and scattered neutrophils (Figure S12 A-C). Inflammatory infiltration extended transmurally through the muscular layer, with prominent lymphoid follicles in the submucosal compartment. Proliferative or regenerative crypts were adjacent to ulcerated regions. Additional sections showed healed ulcerative areas with desquamated mucosal epithelium, inflammatory infiltration between crypts, crypt dilatation, and submucosal edema (Figure S12 D-E). Mild inflammatory cell distribution was randomly observed within the muscular layer, with serosal exudation present. Final sections exhibited healed ulcerative areas appearing as depressed mucosal surfaces with re-epithelialization and regenerative attempts in adjacent crypts (Figure S12 F). Healed regions lacked crypts and showed chronic inflammatory reactions within lamina propria and submucosa, with some dilated crypts present. These findings confirm the successful induction of ulcerative colitis and demonstrate that BRLE mitigated tissue injury in a dose-responsive manner, with the 200 mg/kg dose yielding the most organized histological recovery.


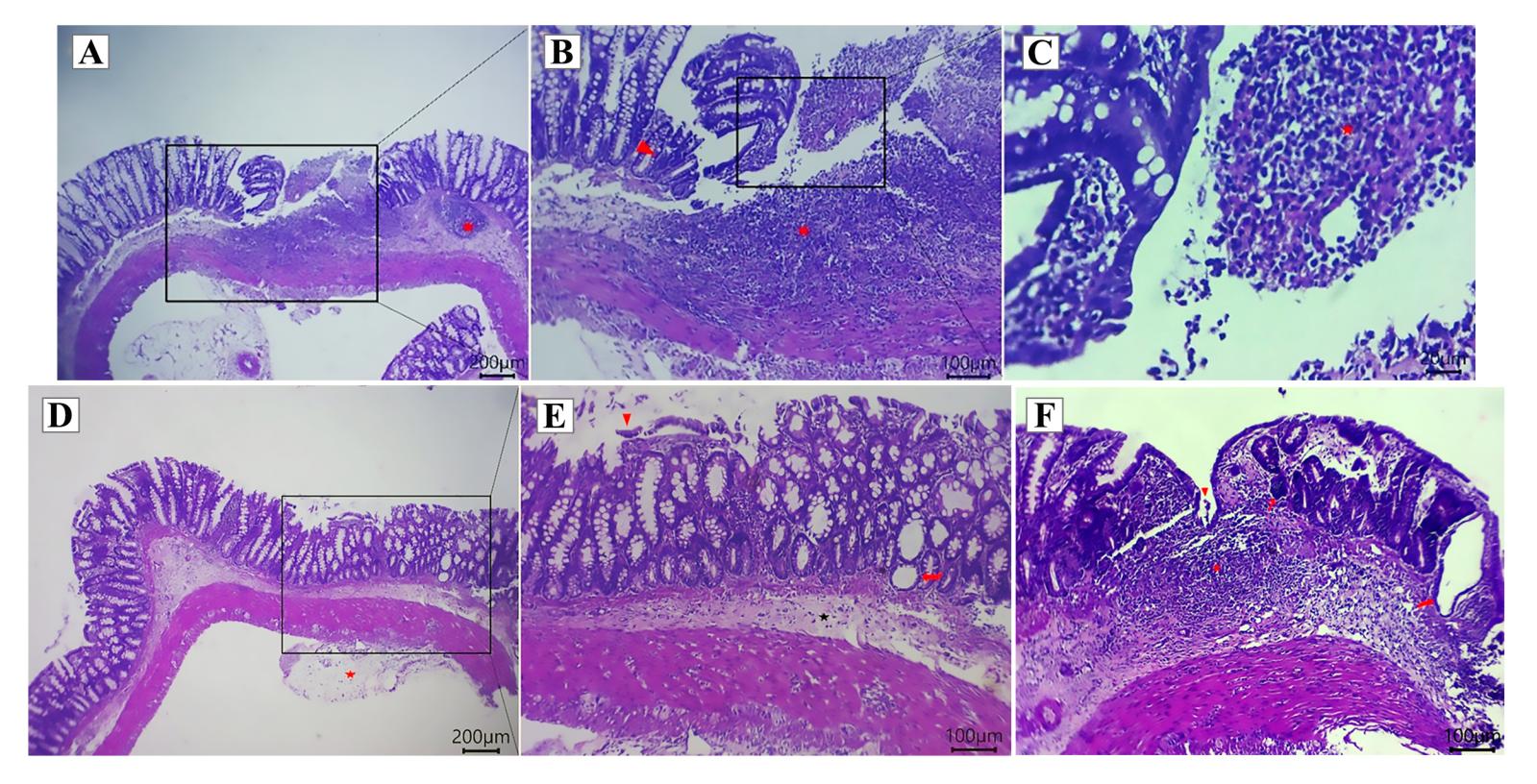


**Figure S12.** Representative H&E-stained colonic sections from the BRLE400-treated group (*Beaucarnea recurvata* leaf extract, 400 mg/kg/day), illustrating ulcerative lesions and healing features. (A–C) Ulcerated mucosa replaced by dense inflammatory cell infiltration (red stars) extending through the full colonic wall, including the muscularis; adjacent regenerating crypts are noted (red arrowhead). (D–E) Healed ulcerated areas with desquamated epithelium (red arrowhead), crypt dilatation (curved red arrow), submucosal edema (black star), and mild inflammatory infiltration of the muscularis with serosal exudation (red star). (F) Re-epithelialization over a healed ulcer, with residual desquamated epithelium (red arrowhead), regenerating crypts (thin red arrow), and areas lacking crypts replaced by chronic inflammatory infiltrates (red star); dilated crypts also observed (curved red arrow). Scale bars: 200 μm, 100 μm, 20 μm.
