# Supplementary material for: Therapeutic Potential of Beaucarnea recurvata Leaf Extract Against Ulcerative Colitis: Integrating Phytochemical Profiling, Network Pharmacology, and Experimental Validation
Source: Int J Mol Sci. 2025 Dec 15;26(24):12053. doi: 10.3390/ijms262412053 (PMC12733345; doi:10.3390/ijms262412053)
Supplement: Supplementary file 1 [file ijms-26-12053-s001.zip › Table S13-16.docx]

**Table S13** Molecular docking interactions between the top-ranked ligands and the EGFR protein, including docking scores, interaction types, involved residues, and corresponding bond distances.

| Ligand | Docking Score (Kcal/mol) | Interaction Type | Residue | Bond Distance (Å) |
| --- | --- | --- | --- | --- |
| Oleanolic acid | −8.80 | Conventional H-bond | MET793 | 2.15 |
| Lucidenic acid A | −8.30 | Conventional H-bond | LYS745 | 3.18 |
|  |  |  | ARG841 | 2.79 |
|  |  |  | LYS875 | 2.34 |
|  |  | Carbon H-bond | GLY721 | 3.51 |
|  |  |  | PRO877 | 3.50 |
|  |  | Unfavorable donor-donor | ARG858 | 2.60 |
|  |  | π-Sigma | PHE856 | 3.63 |
| Hesperetin | −8.10 | Conventional H-bond | ALA722 | 2.91 |
|  |  |  | PHE723 | 3.14 |
|  |  |  | LYS745 | 2.86 |
|  |  |  | ASP837 | 2.25 |
|  |  | π-Sulfur | CYS797 | 5.94 |
|  |  | π-π Stacked | PHE856 | 4.77 and 4.83 |
|  |  | Alkyl | CYS797 | 3.93 |
|  |  |  | LEU844 | 5.26 |
|  |  | π-alkyl | ALA722 | 5.46 |
| Acacetin | −8.10 | Conventional H-bond | ALA722 | 3.01 |
|  |  |  | LYS745 | 2.89 |
|  |  |  | ASP837 | 2.19 |
|  |  | Unfavorable donor-donor | LYS745 | 2.66 |
|  |  | π-Sulfur | CYS797 | 5.96 |
|  |  | π-π Stacked | PHE856 | 3.78, 4.75, and 4.75 |
|  |  | Alkyl | CYS797 | 5.96 |
|  |  |  | LEU844 | 5.32 |
|  |  | π-alkyl | ALA722 | 5.47 |
| 3,9-Dihydroeucomin | −7.80 | Unfavorable donor-donor | ASN842 | 2.63 |
|  |  | π-π Stacked | PHE856 | 3.87 |
|  |  | Alkyl | LEU718 | 5.07 |
|  |  |  | ALA743 | 4.10 |
|  |  |  | LEU792 | 5.26 |
|  |  |  | LEU844 | 5.13 |
|  |  | π-Alkyl | LEU718 | 4.67 |
|  |  |  | VAL726 | 4.54 |

**Table S14.** Molecular docking interactions between the top-ranked ligands and the SRC protein, including docking scores, interaction types, involved residues, and corresponding bond distances.

| Ligand | Docking Score (Kcal/mol) | Interaction Type | Residue | Bond Distance (Å) |
| --- | --- | --- | --- | --- |
| Lucidenic acid A | −9.1 | Conventional H-bond | SER86 | 2.06 |
|  |  |  | ASP148 | 1.93 |
|  |  | Carbon H-bond | ASP148 | 2.94 |
| Oleanolic acid | −7.40 | Conventional H-bond | ILE80 | 2.31 |
| Pinocembrine | −8.20 | Conventional H-bond | GLU54 | 2.1 |
|  |  |  | MET58 | 3.1 |
|  |  | π-π Stacked | TYR84 | 5.51 |
|  |  | π-Alkyl | ALA37 | 4.09 |
|  |  |  | VAL67 | 5.12 |
|  |  |  | LEU137 | 5.07 |
|  |  |  | ALA147 | 4.59 |
| Acacetin | −8.10 | Conventional H-bond | MET58 | 2.60 |
|  |  | Carbon H-bond | ALA147 | 2.78 |
|  |  | π-π Stacked | TYR84 | 5.64 |
|  |  | Alkyl | LEU17 | 4.37 |
|  |  | π-Alkyl | VAL25 | 5.33 |
|  |  |  | ALA37 | 4.28 and 4.90 |
|  |  |  | VAL67 | 4.55 |
|  |  |  | LEU137 | 3.06 |
|  |  |  | ALA147 | 2.78 |
| Hesperetin | −8.10 | Conventional H-bond | MET58 | 2.75 |
|  |  |  | MET85 | 2.17 and 2.73 |
|  |  |  | ASP148 | 2.22 |
|  |  | Carbon H-bond | GLY88 | 2.39 |
|  |  | Alkyl | LEU17 | 4.17 |
|  |  | π-alkyl | VAL25 | 5.27 |
|  |  |  | ALA37 | 4.75 |
|  |  |  | LYS39 | 5.30 |
|  |  |  | LEU137 | 4.91 |

**Table S15.** Molecular docking interactions between the top-ranked ligands and the STAT3 protein, including docking scores, interaction types, involved residues, and corresponding bond distances.

| Ligand | Docking Score (Kcal/mol) | Interaction Type | Residue | Bond Distance (Å) |
| --- | --- | --- | --- | --- |
| Hesperetin | −8.40 | Conventional H-bond | LYS226 | 2.53 |
|  |  |  | LEU285 | 2.34 |
|  |  | Unfavorable donor-donor | THR287 | 1.81 |
|  |  | Alkyl | LEU234 | 4.36 |
|  |  | π-Alkyl | ALA233 | 4.34 |
|  |  |  | LEU285 | 4.15 |
|  |  |  | VAL337 | 4.06 |
| Pinocembrine | −8.30 | Conventional H-bond | ASP225 | 2.57 |
|  |  |  | LEU234 | 2.06 |
|  |  |  | SER237 | 2.59 |
|  |  |  | LEU285 | 2.22 |
|  |  | Carbon H-bond | HIS284 | 2.48 and 2.59 |
|  |  | π-Alkyl | ALA233 | 4.52 and 5.34 |
|  |  |  | VAL337 | 4.47 |
| Acacetin | −8.00 | Conventional H-bond | LEU285 | 2.30 |
|  |  | Carbon H-bond | VAL231 | 3.64 |
|  |  |  | HIS284 | 2.30 and 2.58 |
|  |  | π-Sigma | VAL337 | 2.61 |
|  |  | Alkyl | VAL231 | 5.12 |
|  |  | π-Alkyl | ALA233 | 4.51, 4.57 and 5.25 |
|  |  |  | VAL337 | 4.45 |
| Kaempferol | −7.80 | Conventional H-bond | SER237 | 2.29 |
|  |  |  | LEU285 | 2.42 |
|  |  | Carbon H-bond | HIS284 | 2.58 and 2.64 |
|  |  | π-Sigma | VAL337 | 2.53 |
|  |  | π-Alkyl | ALA233 | 4.47, 4.60 and 5.04 |
|  |  |  | VAL337 | 4.42 |
| Pechueloic acid | −7.50 | Conventional H-bond | SER228 | 2.44 and 2.73 |
|  |  | Carbon H-bond | SER237 | 2.76 |
|  |  | Alkyl | ALA233 | 4.87 |
|  |  |  | LEU285 | 5.37 |
|  |  |  | VAL337 | 3.86 |

**Table S16.** Molecular docking interactions between the top-ranked ligands and the AKT1 protein, including docking scores, interaction types, involved residues, and corresponding bond distances.

| Ligand | Docking Score (Kcal/mol) | Interaction Type | Residue | Bond Distance (Å) |
| --- | --- | --- | --- | --- |
| Lucidenic acid A | −8.80 | Conventional H-bond | LYS36 | 2.11 and 5.01 |
|  |  |  | LYS133 | 2.42 |
|  |  | Carbon H-bond | LYS36 | 2.58 |
| Hesperetin | 8.50 | Conventional H-bond | LYS15 | 2.70 |
|  |  |  | ASP296 | 2.75 |
|  |  | π-Anion | GLU91 | 3.21 |
|  |  | π-Sigma | VAL21 | 2.82 |
|  |  | π-Sulfur | MET138 | 3.76 |
|  |  | Alkyl | MET84 | 3.88 |
|  |  | π-Alkyl | ALA34 | 4.23 |
| Pinocembrine | −8.20 | Conventional H-bond | LYS15 | 3.06 |
|  |  |  | GLU91 | 2.95 |
|  |  | π-Anion | GLU91 | 3.25 |
|  |  | π-Sulfur | MET138 | 3.77 |
|  |  | π-Alkyl | LEU13 | 5.32 |
|  |  |  | VAL21 | 4.69 |
|  |  |  | ALA34 | 3.85 |
|  |  |  | ALA87 | 5.01 |
| Acacetin | −8.10 | Conventional H-bond | LYS15 | 2.76 |
|  |  | π-Anion | GLU91 | 3.19 and 3.92 |
|  |  | π-Sigma | VAL21 | 2.87 |
|  |  | π-Sulfur | MET138 | 3.75 |
|  |  | Alkyl | MET84 | 3.83 |
|  |  | π-Alkyl | VAL21 | 4.83 |
|  |  |  | ALA34 | 4.46 |
| 3,9-Dihydroeucomin | −8.00 | Conventional H-bond | ASP296 | 2.11 |
|  |  | Carbon H-bond | GLU91 | 3.46 |
|  |  |  | PHE295 | 2.89 |
|  |  | π-Anion | GLU91 | 3.48 |
|  |  | π-Sulfur | MET138 | 3.76 |
|  |  | π-Alkyl | VAL21 | 4.23 |
|  |  |  | ALA34 | 4.85 |
|  |  |  | PHE91 | 4.97 |
