# Supplementary material for: Therapeutic Potential of Beaucarnea recurvata Leaf Extract Against Ulcerative Colitis: Integrating Phytochemical Profiling, Network Pharmacology, and Experimental Validation
Source: Int J Mol Sci. 2025 Dec 15;26(24):12053. doi: 10.3390/ijms262412053 (PMC12733345; doi:10.3390/ijms262412053)
Supplement: Supplementary file 1 [file ijms-26-12053-s001.zip › Table S17.docx]

**Table S17.** Target proteins, and the corresponding grid coordinates.

| Target protein | PDB ID | Grid box coordinates | |
| --- | --- | --- | --- |
|  |  | **Centers (x, y, z)** | **Sizes (x, y, z)** |
| EGFR | 5U8L | 3.09465, -13.4152, -37.878 | 33.4942, 34.9322, 34.2797 |
| SRC | 4MXO | 8.8662, -34.5176, -11.3427 | 28.3101, 28.3873, 29.2825 |
| STAT3 | 6QHD | -31.9451, -38.9162, 2.08569 | 31.4268, 30.1364, 45.6048 |
| AKT1 | 4EKL | 27.1126, 5.5672, 13.7917 | 17.6017, 24.3212, 17.5834 |
