# Supplementary material for: Therapeutic Potential of Beaucarnea recurvata Leaf Extract Against Ulcerative Colitis: Integrating Phytochemical Profiling, Network Pharmacology, and Experimental Validation
Source: Int J Mol Sci. 2025 Dec 15;26(24):12053. doi: 10.3390/ijms262412053 (PMC12733345; doi:10.3390/ijms262412053)
Supplement: Supplementary file 1 [file ijms-26-12053-s001.zip › Table S18.docx]

**Table S18.** Macroscopic pathological scoring system for colonic inflammation.

| **Score** | **Macroscopic Observation** |
| --- | --- |
| **0** | No observable abnormalities |
| **1** | Mucosal hyperemia only |
| **2** | Mild mucosal edema with minimal bleeding or superficial erosions |
| **3** | Moderate edema, hemorrhage, and ulcerative lesions |
| **4** | Extensive ulceration with severe edema and tissue necrosis |
