# Supplementary material for: Therapeutic Potential of Beaucarnea recurvata Leaf Extract Against Ulcerative Colitis: Integrating Phytochemical Profiling, Network Pharmacology, and Experimental Validation
Source: Int J Mol Sci. 2025 Dec 15;26(24):12053. doi: 10.3390/ijms262412053 (PMC12733345; doi:10.3390/ijms262412053)
Supplement: Supplementary file 1 [file ijms-26-12053-s001.zip › Table S7-S8.docx]

**Table S7.** Core protein targets identified via PPI network analysis with corresponding topological centrality parameters (BC: betweenness centrality; CC: closeness centrality; DC: degree centrality).

| Target name | BC | CC | DC |
| --- | --- | --- | --- |
| EGFR | 6789.466 | 0.125874 | 80 |
| SRC | 5361.891 | 0.124745 | 74 |
| AKT1 | 6624.905 | 0.125978 | 73 |
| STAT3 | 5186.433 | 0.125153 | 73 |
| HSP90AA1 | 5742.358 | 0.123786 | 68 |
| TNF | 5919.774 | 0.124441 | 58 |
| BCL2 | 3600.473 | 0.123537 | 57 |
| JUN | 2070.701 | 0.124037 | 52 |
| CASP3 | 2517.707 | 0.123337 | 50 |
| ESR1 | 4980.534 | 0.123586 | 50 |
| ERBB2 | 2494.003 | 0.12201 | 45 |
| STAT1 | 914.7756 | 0.122156 | 45 |
| PTGS2 | 9413.514 | 0.123387 | 44 |
| NFKB1 | 1501.716 | 0.123139 | 44 |
| CCND1 | 1558.273 | 0.121525 | 43 |
| MAPK3 | 2199.264 | 0.122156 | 42 |
| MAPK1 | 2047.809 | 0.122253 | 41 |
| TLR4 | 2222.775 | 0.122892 | 41 |
| MMP9 | 1845.109 | 0.122547 | 39 |
| CXCL8 | 4192.256 | 0.121815 | 38 |
| HDAC1 | 1361.373 | 0.119578 | 36 |
| PARP1 | 2178.028 | 0.12 | 34 |
| FOS | 974.423 | 0.121525 | 33 |
| ALB | 5291.236 | 0.122744 | 32 |

**Table S8.** Ranked Beaucarnea recurvata leaf extract compounds based on degree centrality (DC) analysis.

| Rank | Name | Score |
| --- | --- | --- |
| **1** | Lucidenic acid A | 81 |
| **2** | 3,9-Dihydroeucomin | 76 |
| **3** | Scaposin | 72 |
| **4** | 3-Methoxynobiletin | 71 |
| **5** | Acacetin | 69 |
| **6** | Kaempferol | 68 |
| **7** | Pinocembrine | 68 |
| **8** | Pechueloic acid | 63 |
| **9** | Hesperetin | 60 |
| **10** | Oleanolic acid | 56 |
| **11** | Ursolic acid | 55 |
| **12** | Sinapaldehyde | 49 |
| **13** | Umbelliferone | 47 |
| **14** | Ruscogenin | 36 |
| **15** | Eucomol | 36 |
| **16** | Caffeic acid | 36 |
| **17** | sebacic acid | 31 |
| **18** | Octanedioic acid | 25 |
| **19** | p-hydroxylcinnamaldehyde | 24 |
| **20** | Gallic acid | 13 |
| **21** | 4-hydroxyphenylacetic acid | 12 |
| **22** | 2,3-Dihydroxybenzoic acid | 11 |
| **23** | 4'-Hydroxyacetophenone | 9 |
| **24** | Aucubin | 9 |
| **25** | 3,4-Dihydroxybenzaldehyde | 8 |
| **26** | Methyl 3,4-dihydroxybenzoate | 0 |
| **27** | p-coumaric acid | 0 |
| **28** | azelaic acid | 0 |
| **29** | salicylic acid | 0 |
